# A Systematic Review of Synthetic and Anticancer and Antimicrobial Activity of Quinazoline/Quinazolin‐4‐one Analogues

**DOI:** 10.1002/open.202400439

**Published:** 2025-01-28

**Authors:** Neha Manhas, Gobind Kumar, Sanjeev Dhawan, Talent Makhanya, Parvesh Singh

**Affiliations:** ^1^ Department of Chemistry Durban University of Technology ML Sultan Campus Durban 4000 South Africa; ^2^ School of Chemistry and Physics University of KwaZulu-Natal P/Bag X54001 Westville, Durban 4000 South Africa

**Keywords:** Quinazoline, Quinazolin-4-one, Metal catalyst, Metal-free, Miscellaneous reagents, Antibacterial, Anticancer, Antifungal

## Abstract

Quinazolines/quinazolin‐4‐ones are significant nitrogen‐containing heterocycles that exist in various natural products and synthetic scaffolds with diverse medicinal and pharmacological applications. Researchers across the globe have explored numerous synthetic strategies to develop safer and more potent quinazoline/quinazolinone analogues, particularly for combating cancer and microbial infections. This review systematically examines scholarly efforts toward understanding this scaffold's synthetic pathways and medicinal relevance, emphasizing the role of metal and non‐metal catalysts and other reagents in their synthesis. Additionally, the article discusses selected compounds’ anticancer and antimicrobial properties, with a brief look into their structure‐activity relationships.

## Graphical abstract

Quinazoline represents an important class of heterocycles with diverse medicinal and pharmacological significance. This review systematically examines scholarly efforts toward understanding different synthetic pathways emphasizing the role of metal and non‐metal catalysts including some miscellaneous reagents employed in the synthesis of quinazoline analogues. Furthermore, the article discusses selected compounds′ anticancer and antimicrobial properties, with a brief look into their structure‐activity relationships.

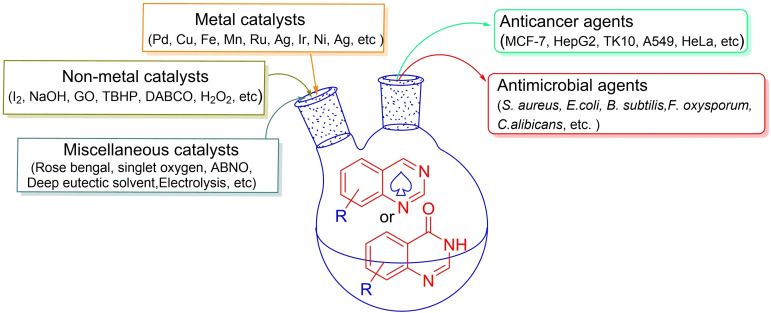

## 1 Introduction

The world of today has awoken to a dawn of extreme call for action as a result of an ongoing increase in mortality rates and excess medical care costs. This is due to the relentless evolution of pathogenic microbes and their corresponding resistance to available therapeutic agents. Hence, the phrase ‘antimicrobial resistance’ has become the epicenter of many medicinal chemistry and public health reports and colloquiums. Further, available reports and statistics from different agencies on the subject matter have continued to elicit unrest in the heart of researchers in the field.

For instance, a report by the Centres for Disease Control and Prevention (CDC) highlighted the severity and threat level of eighteen antibiotic‐resistant bacteria strains.^[1]^ In the report, diarrhoea‐causing bacteria – *Clostridium difficule* is classified as an urgent threat responsible for an estimated 15,000 deaths and almost half a million infections among patients in the United States annually with a possible $3.8 billion savings in medical costs over five years if the pathogen is contained. Fluconazole‐resistant *Candida*, the fungus responsible for Candidemia is classified as a serious threat as a result of the increase in *Candida* infections (46,000 infections per year) due to azole and echinocandin‐resistant strains, especially in immune‐compromised patients. Carbapenem‐resistant *Enterobacteriacea* is said to be evasive to all or nearly all available antibiotic agents, hence regarded as an urgent threat. In addition, the World Health Organization (WHO) reported the severity of the resistance levels of Gram‐negative bacteria; *Enterobacteriaceae, Pseudomonas aeruginosa*, and *Acinetobacter*, to carbapenems and cephalosporins, the best available antibiotics for treating multi‐drug resistant bacteria, hence requiring urgent research and development (R&D) attention (WHO 2023).^[2]^

However, with these unnerving statistics, there has been a decline in antimicrobial R&D as seen in the number of new antimicrobial drugs approved over the past decade in the face of an unleashing of new resistant strains from the microbial world.^[3]^ These are attributable to; (i) the withdrawal of big pharmaceutical companies from antimicrobial research,^[4]^ which in turn affects funding available for research by academia, (ii) strict regulatory protocols for the approval of antimicrobial agents,^[5]^ (iii) greater financial returns in other therapeutic fields such as oncology^[6]^ and (iv) the misuse and abuse of available antimicrobials thus fostering the development of resistant mechanisms by the microbes.

The tendency of microorganisms, including bacteria, to develop resistance is an inevitable process that occurs through a battery of defensive mechanisms and enzymatic activities, encumbering the efficacy of drugs. For example, β ‐lactamases in bacteria, hydrolytically cleave the lactam ring of several β ‐lactam‐derived antibiotics (e. g. cephalosporins), making them inactive. Nevertheless, egress from the persisting crisis without going in circles is solely based on the development of new antimicrobial agents with new mechanisms of action and a disciplined policy on their utility.

**Figure 1:**
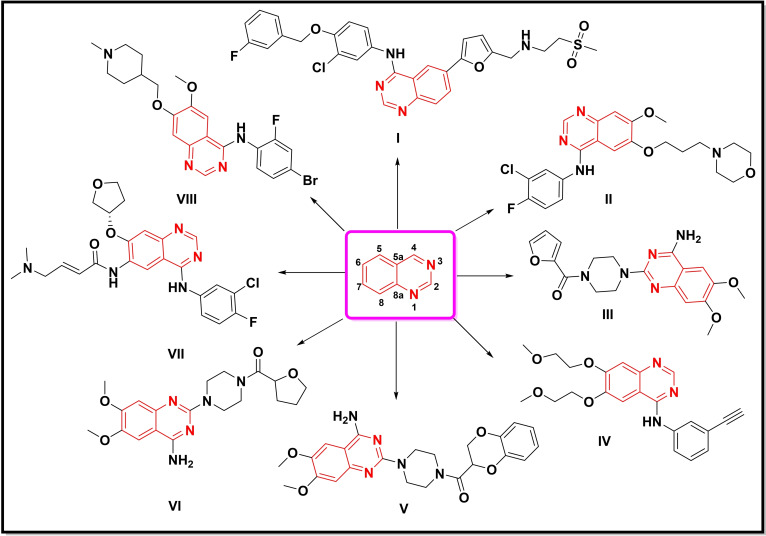
Quinazoline‐bearing bioactive compounds and commercial FDA‐approved drugs.

Quinazoline is a heterobicyclic compound in which a benzene ring is fused to a pyrimidine ring (Figure 1). The parent quinazoline was synthesized by Gabriel *et al*. in 1903 using *o*‐nitroaniline as a precursor, whereas its natural analogue was isolated from the Chinese plant aseru. Even since, a plethora of heterocyclic compounds bearing quinazoline moiety as their key structural unit has been widely synthesized and documented in the literature.

The quinazoline ring represents a class of fused heterocycles with a wide‐spectrum of biological and pharmacological activities including, antimicrobial, anti‐tubercular, antiviral, anti‐tumor, anti‐malarial, anti‐depressant, anti‐inflammatory and anti‐histaminic activity.[^7^, ^8^, ^9^, ^10^] A variety of existing drugs, for example, lapatinib **I** (tyrosine kinase inhibitor and antineoplastic agent), gefitinib **II** (anticancer), prazosin **III** (antihypertension), erlotinib **IV** (anticancer), doxazosin **V** (high blood pressure), terazosin **VI** (antihypertensive), afatinib **VII** (anti‐inflammatory), and vandetanib **VIII** (anticancer) also contain quinazoline ring in their core structure[^8^, ^10^, ^11^] Consequently, the development of new synthetic strategies for the preparation of new quinazoline analogues has received considerable attention among synthetic and medicinal chemists worldwide.

**Figure 2:**
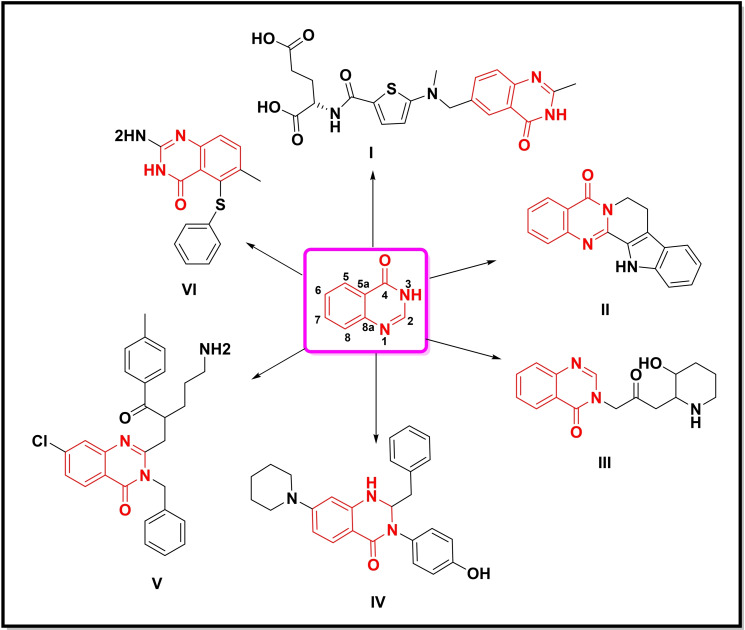
Potent compounds containing quinazolin‐4‐one moiety.

Quinazolin‐4‐one, a structural analogue of quinazoline bearing a keto functionality at position 4, is also an important scaffold with a wide spectrum of biological activities including, anticancer, antimicrobial, anti‐inflammatory, and tyrosine kinase inhibitor, etc. Some potent compounds bearing quinazolin‐4‐one moiety utilized in the market as anticancer agents (**I**, **II**, **VI**) and kinase inhibitors (**III**, **IV**, **V**) are depicted in Figure 2.^[8]^

The most commonly employed synthetic strategy for the preparation of quinazoline architecture utilizes anthranilic acid and its carbonyl counterparts such as formamide,[^12^, ^13^, ^14^] acid chlorides,[^15^, ^16^] urea,^[17]^ and aldehydes,[^18^, ^19^] as starting materials under dehydrating conditions. In some cases, the coupling/cyclization reaction of amidines with 2‐bromobenzonitriles^[20]^ or the nucleophilic aromatic substitution/cyclization reactions of 2‐fluorobenzonitriles with benzonitriles have also been engaged in the synthesis of 4‐substituted quinazolines.^[21]^ Additionally, the role of iodine[^13^, ^15^] and different metals such as copper[^22^, ^23^] have also been demonstrated in quinazoline synthesis.

The following sections cover the relevant literature published in the past eight years (2016–2023) focusing on various reaction pathways for the synthesis of quinazolines and quinazolin‐4‐ones achieved in the presence/absence of metal catalysts including some miscellaneous reagents. In addition, the anticancer and antimicrobial activities of some representative are also described. Although the synthetic and medicinal applications of quinazoline analogues have already been reviewed by different authors in the recent past few years,[^24^, ^25^, ^26^, ^27^, ^28^, ^29^, ^30^, ^31^] this is the first article in which metal/non‐metal catalysed synthesis of this excellent scaffold has been systematically discussed including some miscellaneous catalysts along with their anticancer and antimicrobial activities.

## 2 Literature Study

The following sections contain a brief discussion on the synthesis of quinazoline/quinazolin‐4‐one derivatives in the presence of metals followed by the application of miscellaneous reagents and then metal catalysts. The biological activities of the aforementioned heterocycles are subsequently discussed followed by a tabulated summary of all papers for a quick recap.

### 2.1 Metal Catalysed Synthesis

**Scheme 1:**
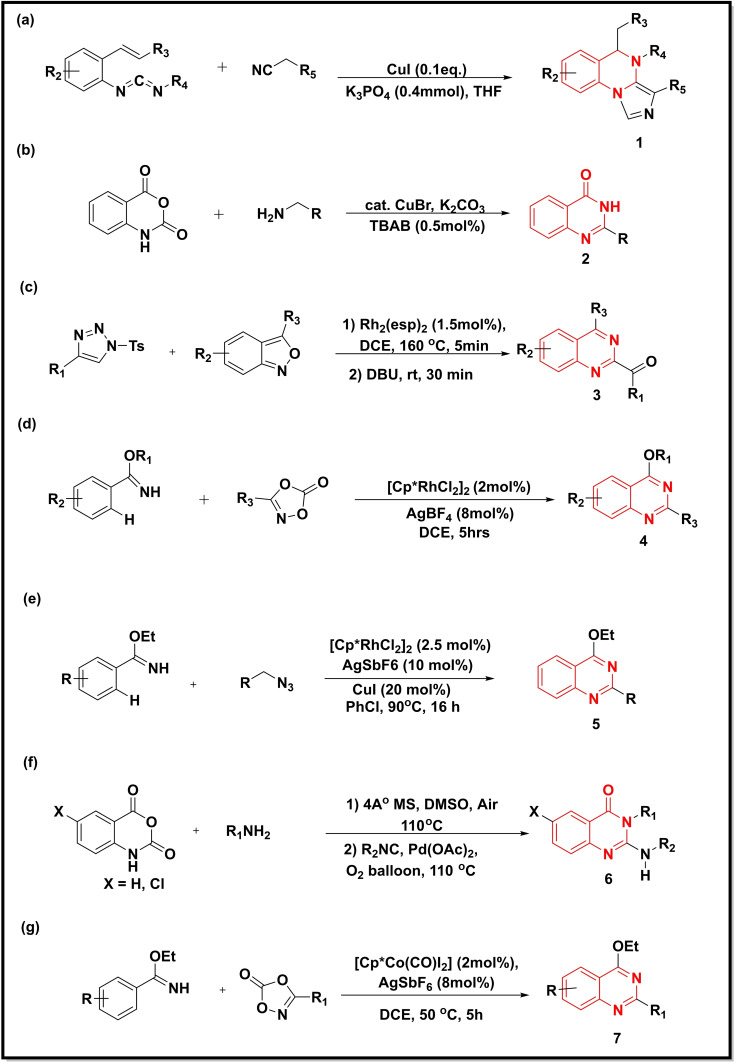
Synthesis of quinazoline‐4‐ones/quinazolines from carbodiimides, isatoic anhydrides, benzimidates and substituted triazoles.

Synthesis of imidazo‐quinazolines (**1**) was carried out *via* a cascade reaction of *o*‐alkenylphenyl carbodiimides with isocyanides using copper as a catalyst (Scheme 1a).^[32]^ All these reactions were reported to be facilitated by the use of either a Lewis acid or a Lewis base. The reaction mechanism initially involved the intermediacy of a [3+2] cycloaddition reaction followed by an intramolecular annulation reaction. Another one‐pot synthesis of 2‐substituted quinazoline‐4‐ones (**2**) was performed from the reaction of substituted arylmethanamines with isatoic anhydride in the presence of copper bromide (Scheme 1b). The reaction proceeded *via* an *in situ* oxidation‐cyclization reaction affording the quinazoline‐4‐one derivatives in good yields.^[23]^ Rhodium (Rh(II)) catalysed and DBU‐promoted transannulation synthesis of 2,4‐disubstituted quinazoline derivatives (**3**) was developed using *N*‐sulfonyl‐1,2,3‐triazoles and different substituted 1,2‐benzisoxazoles as starting materials (Scheme 1c). The reaction underwent sequential intramolecular 1,3‐sulfonyl migration, followed by an aza‐[4+2] cycloaddition.^[33]^ Using rhodium as a catalyst, a two‐component reaction sequence for functionalized quinazolines (**4**) was reported from different benzimidates and dioxazolones (Scheme 1d). Optimization studies revealed a catalyst system consisting of [Cp*RhCl_2_]_2_ and AgBF_4_ as the best combination for these conversions. This reaction mechanism involved the C−H activation, cyclisation, and denitration to obtain substituted quinazolines.^[34]^ Similarly, Wang and co‐workers developed an interesting rhodium‐ and copper‐co‐catalyzed route to biologically important quinazolines (**5**), starting from different imidates and alkyl azides (Scheme 1e). A diverse range of quinazolines was successfully synthesized in moderate to good yields using optimized conditions with O_2_ as an oxidant at 90 °C. These transformations involved direct C−H bond activation, intramolecular addition, and aerobic oxidative aromatization.^[35]^ A palladium catalysed and ligand‐free synthesis of 2‐aminoquinazoline‐4‐ones (**6**) was described using a multi‐component reaction (MCR) between different isocyanides and aromatic amines (Scheme 1f) and oxazine analogues. The MCR was successful in introducing different functional groups in the quinazoline core with moderate to good yields.^[36]^ The catalytic performance of different group 9 triads [Cp*M(III)] catalysts (M=Co, Rh, Ir) was examined for the synthesis of quinazolines using ethyl benzimidate and dioxazolones as precursors (**7**).^[37]^ The Cp*Co(III) catalyst offered the best yield (72–93 %) and selectivity, whereas much lower yields were obtained with Cp*Rh(III) and Cp*Ir(III) (Scheme 1g). The reaction mechanism involved consecutive [4+2] cycloadditions, cobalt‐catalyzed C−H amidations, followed by cyclisation.

**Scheme 2:**
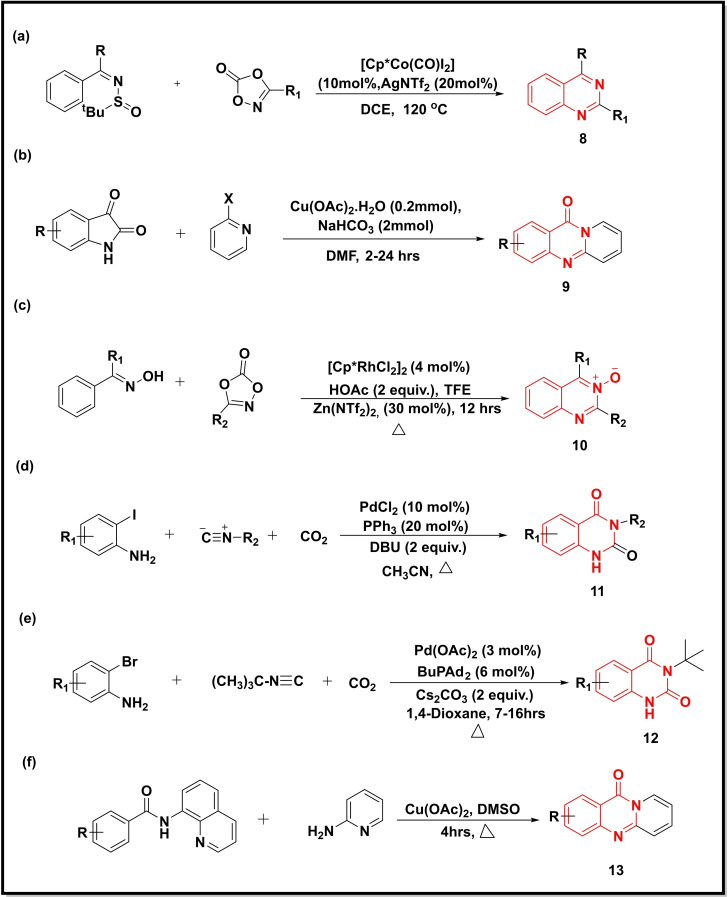
Synthesis of quinazoline‐4‐ones/quinazolines from carbodiimides, isatoic anhydrides, benzimidates and substituted triazoles.

A cobalt catalysed C−H activation of *N*‐sulfinylimines and benzimidates and their coupling with dioxazolones led to a wide range of substituted quinazolines (**8**) in good yields (Scheme 2a). The reaction proceeded with high regio‐, and mono/di‐selectivity. In benzimidates bearing different *ortho*, *meta* and *para* substituents, the reactions worked well with high efficiency and selectivity.^[38]^

Liu *et al*. used (Cu(OAc)_2_⋅H_2_O) for the condensation of isatins and 2‐bromopyridine to synthesise pyrido‐quinazolinones (**9**) (Scheme 2b). The isatin and 2‐bromopyridine both bearing electron‐releasing groups as well as electron‐accepting groups afforded good to excellent yields (75–94 %). However, the presence of a bulky group at 2‐bromopyridine compromised the yield probably attributed to steric hindrance.^[39]^ Wang et al reported the synthesis of quinazoline N‐oxides (**10**) from ketoximes and 1,4,2‐dioxazol‐5‐ones using Rh (III) and Zn (II) catalysts, where each catalyst played a specific role (Scheme 2c). For example, the Rh(III)‐catalyst promoted the amidation of the ketoximes via C−H activation whilst the Zn(II)‐catalyst triggered the cyclization. The presence of functional at the *meta* position shows high regioselectivity of oxime while their positioning at the *ortho* position decreased its regioselectivity due to steric hindrance.^[40]^ A Pd‐catalyzed [PdCl_2_] three‐component reaction between 2‐iodoanilines, isocyanides and carbon dioxide led to the synthesis of N3‐substituted quinazoline‐2,4‐(1H,3H)‐diones (**11**) in moderate to excellent yield (40–96 %) (Scheme 2d).^[41]^ Another study utilized [Pd(OAc)_2_] for the insertion and cycloaddition of CO_2_ and isocyanide with 2‐bromoanilines to furnish N3‐substituted quinazoline‐2,4‐(1H,3H)‐diones (**12**) (Scheme 2e). The reactions were reported to proceed in a chemo‐ and regioselective manner.^[42]^ The Cu‐catalysed synthesis of pyrido‐quinazolinones (**13**) through condensation of 8‐aminoquinoline and 2‐aminopyridine was reported (Scheme 2f). The benzamides bearing electron‐releasing groups (Me and OMe) showed higher reactivity than those bearing electron‐accepting groups (F, Cl, NO_2_, CN, and Ac). The positioning of these substituents to 2‐aminopyridine furnished moderate to good yields (54–78 %).^[43]^

**Scheme 3:**
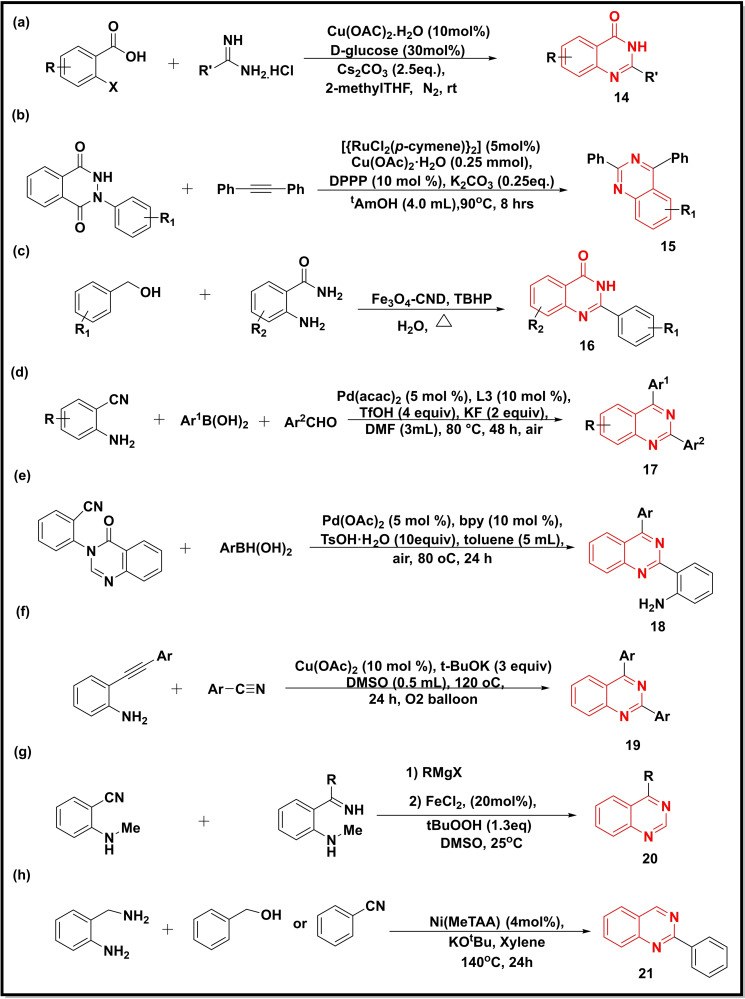
Synthesis of quinazoline‐4‐ones/quinazolines from benzoic acids, benzamides, benzimidates, aminobenzonitriles, and substituted anilines.

A sustainable and environment‐friendly method was reported for the synthesis of quinazolinones (**14**) using 2‐halobenzoic acids and substituted amidines in the presence of Cu(OAc)_2_⋅H_2_O−glucose in a green solvent (2‐methylTHF) (Scheme 3a). Glucose was reported to play a dual role as a chelating agent as well as a reducing agent in the reaction. The scope of this method was further demonstrated by synthesizing diproqualone, an anti‐inflammatory drug.^[44]^ Prakash *et al*. used Ru(II) catalyst to synthesize quinazolines (**15**) in moderate to good yield (50–80 %) starting from 2‐phenyldihydrophthalazinediones and alkynes (Scheme 3b). The reaction proceeded via annulations as well as C−H activation.^[45]^ Another study utilized Fe in the synthesis of magnetic nanocatalyst (Fe_3_O_4_‐CND) which was subsequently used to prepare 2‐substituted quinazolinones **16** (yield 63–94 %) using 2‐amino benzamides and benzyl alcohols as starting materials and water as a green solvent (Scheme 3c).^[46]^ The commercially available substances like aromatic aldehydes, 2‐aminobenzonitrile and arylboronic acids were utilized in the synthesis of 2,4‐disubstituted quinazolines (**17**) using Pd‐catalyst [Pd(acac)_2_] in moderate to good yield (48–91 %) for all the precursors (Scheme 3d).^[47]^ Zhang and coworkers also synthesized 2,4‐disubstituted quinazolines (**18**) via Pd‐catalysed [Pd(OAc)_2_] reaction of arylboronic acids with 2‐(quinazolinone‐3(4*H*)‐yl)benzonitriles (Scheme 3e).^[48]^ A Copper catalyst was used to catalyze the chemical reaction of 2‐ethynylanilines with benzonitriles to afford substituted quinazolines (**19**) (Scheme 3f). The molecular oxygen was used as an oxidant to cleavage the C−C triple bond in this reaction. The benzonitriles bearing EWG gave better yields (67–88 % yield) than those bearing EDG (41–61 %).^[49]^ The N−H ketimines prepared from the corresponding ortho‐alkylamino benzonitriles by the action of Grignard Reagent underwent intramolecular cyclization in the presence of a Fe‐catalyst (FeCl_2_) and *tert‐*BuOOH to afford 4‐substituted quinazolines (**20**) (Scheme 3g).^[50]^ A Ni‐catalyzed synthesis of 2‐substituted quinazolines (**21**) followed two pathways: one involving the condensation of benzyl alcohol with 2‐aminobenzylamine while the second involving the condensation between 2‐aminobenzylalcohol and benzonitrile (Scheme 3h).^[51]^

**Scheme 4:**
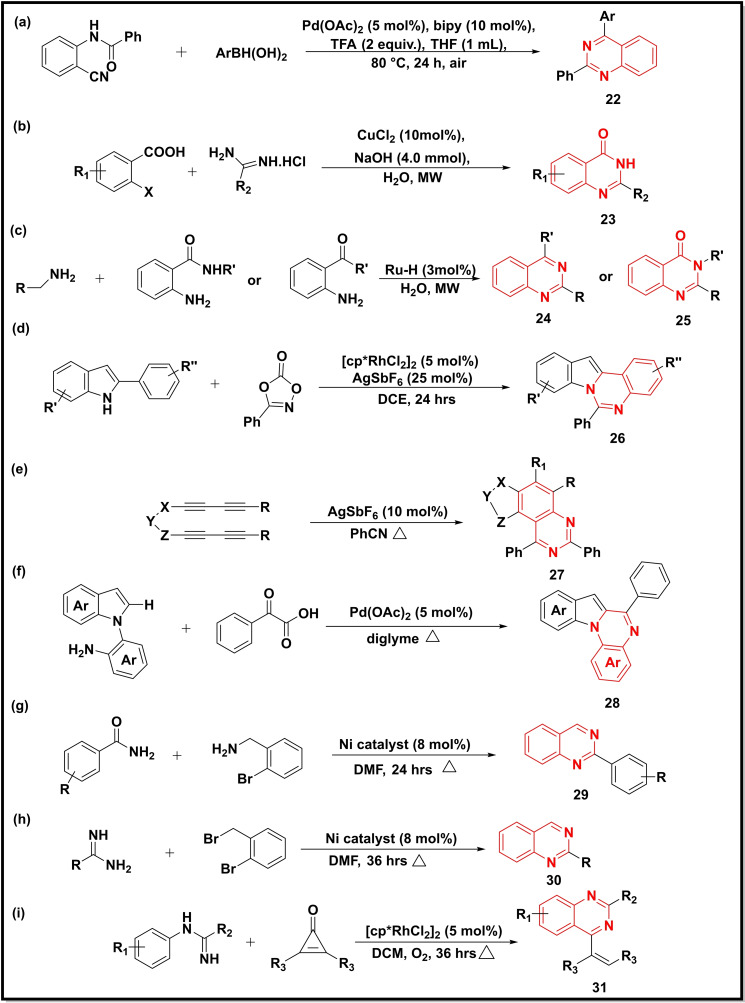
Synthesis of quinazoline‐4‐ones/quinazolines from benzoic acids, amidines, benzamides, alkynes, aminobenzonitriles, substituted anilines, and indoles.

Another methodology afforded substituted quinazolines (**22**) in good yields (51–94 %) *via* Pd‐catalyzed [(Pd(OAc)_2_] cyclization of N‐(2‐cyanoaryl)benzamides using arylboronic acids (Scheme 4a). The strategy was reported to show reasonable functional group tolerance ability of hydroxyl and halogen substituents.^[52]^ The Cu‐catalysed coupling between amidines and 2‐halobenzoic acids under microwave energy led to quinazolinones (**23**) in water (Scheme 4b). The reaction involving iodobenzoic acid displayed higher reactivity than its 2‐Cl and 2‐Br substituted analogs. Also, higher yields were obtained when EWG groups (e. g. 4‐NO_2_=94 %) were present on the benzene ring.^[53]^ Arachchige *et al*. reported the coupling of 2‐aminobenzamides and 2‐ aminophenyl ketones with amines to synthesise substituted quinazolines (**24** and **25**) (Scheme 4c). The reaction proceeded via deamination and then dehydrogenation. The in‐situ generated Ru−H complex with a ligand (catechol) catalyzed this reaction and was reported to induce selectivity in these reactions.^[54]^ The Rh‐catalyzed [Cp*RhCl_2_]_2_ intramolecular cyclization and amidation of 2‐aryl‐1H‐indoles with dioxazolone afforded indolo[1,2‐c]quinazolines (**26**) in good yields (55–95 %) (Scheme 4d). The mechanistic cycle indicated that AgSbF_6_ and LiOAc activate the Rh‐catalyst.^[55]^ Similarly, the silver catalyst (AgSbF_6_) triggered the [A+2B] annulation of arynes with nitriles to generate polysubstituted quinazolines (**27**) (Scheme 4e). The density function theory calculations indicated the intermediacy of the nitrilium ion in the reaction.^[56]^ Synthesis of indolo[1,2‐ *a*]quinazolines (**28**) was achieved using Pd as a catalyst and indols and *α*‐oxocarboxylic acids as starting reagents (Scheme 4f). The reaction proceeded with good regioselectivity offering the final compounds in moderate to good yield.^[57]^ Another methodology derived the 2‐substituted qunazolines (**29** and **30**) via a cross‐coupling reaction of amidines and benzamides with 2‐bromobenzylbromide and 2‐bromobenzylamine, respectively using Ni‐catalyst (Schemes 4g and h).^[58]^ The usage of Rh(III)‐catalyst [Cp*RhCl_2_]_2_ afforded 4‐ethenyl quinazolines (**31**) starting from cyclopropenones and *N*‐arylamidines (Scheme 4i). The mild reaction protocols, atom economy and neutral byproducts were some features of the reaction protocol.^[59]^

**Scheme 5:**
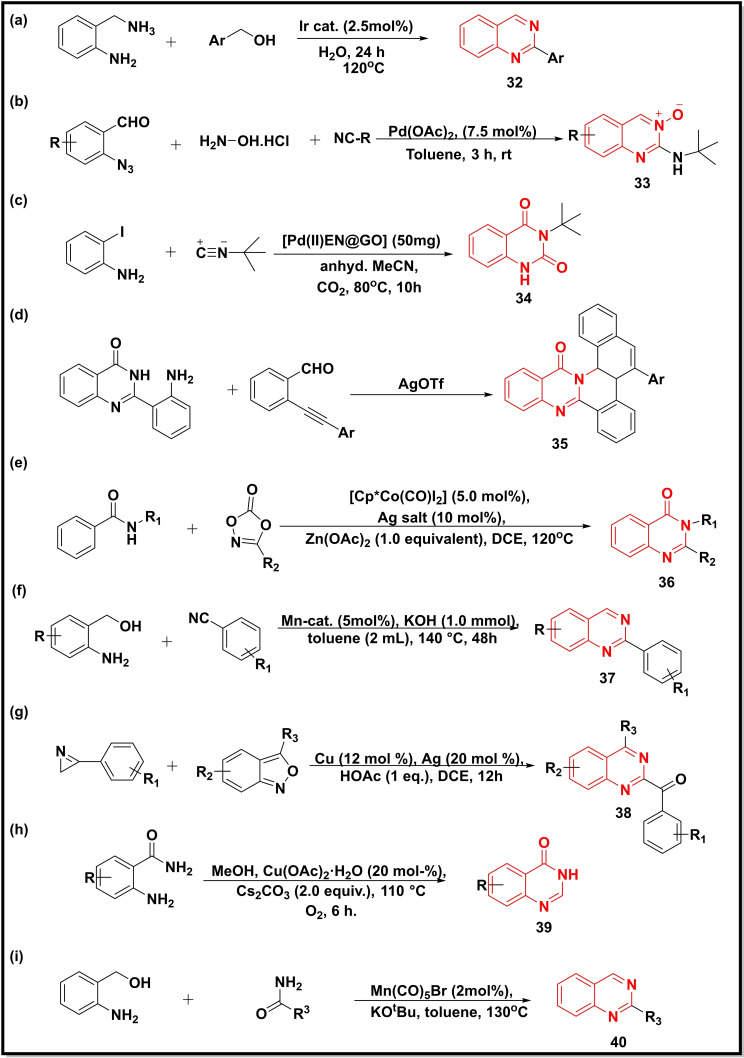
Synthesis of quinazoline‐4‐ones/quinazolines from arylamines, aryl azides, benzamides, and aziridines.

The Ir catalyst was employed in the synthesis of 2‐substituted quinazolines (**32**) (68–94 %) via coupling of 2‐aminobenzylamine with primary alcohols (Scheme 5a). The aromatic alcohols bearing both EDG as well as EWG afforded good yields including hetero‐aromatic alcohols (68–78 %).^[60]^ The Pd(OAc)_2_ catalyst promoted a multicomponent reaction between isocyanides, 2‐azidobenzaldehydes, and hydroxylamine hydrochloride to form quinazoline 3‐oxides (**33**) in moderate to high yield (Scheme 5b).^[61]^ In another work, [Pd(II)EN@GO] catalyst was used to synthesize quinazoline‐dione (**34**) in good to excellent yield (55–94 %) using 2‐iodoaniline, isocyanides and CO_2_ as the precursors (Scheme 5c).^[62]^ Another reaction strategy achieved the substituted quinazoline‐4‐ones (**35**) in good yields (79–89 %) by reacting 2‐(2‐aminophenyl)quinazolin‐4(3*H*)‐ones and 2‐(2‐arylethynyl) benzaldehydes in the presence of AgOTf catalyst (Scheme 5d).^[63]^ A Cp*CoIII‐catalyzed reaction afforded 2‐alkyl‐substituted quinazolinones (**36**) via C−H bond annulation/amidation of benzamides with oxazole (Scheme 5e). The reaction was reported to exhibit a broad scope of substrate with moderate to good yield.^[64]^ Mn‐catalyst was employed to achieve the condensation of 2‐amino benzyl alcohols with benzonitriles to furnish 2‐substituted quinazolines (**37**) in good yields (Scheme 5f).^[65]^ The Cu/Ag‐catalyzed coupling reaction between 3‐aryl‐2H‐azirines and anthranils led to the formation of 2,4‐disubstituted quinazolines (**38**) in moderate to good yields (Scheme 5g). The mechanistic pathway indicated that copper promotes the cleavage of the N−O bond of anthranil and the N−C2 bond of azirine.^[66]^ The reaction of 2‐aminobenzamide with methanol in the presence of Cu‐catalyst [Cu(OAc)_2_] afforded quinazoline‐4‐ones (**39**) in good to excellent yield (up to 99 %) (Scheme 5h).^[67]^ Mn‐catalyst was explored for the synthesis of quinazolines (**40**) via acceptorless dehydrogenative coupling between 2‐aminobenzyl alcohol and benzamides in the absence of any additional oxidant (Scheme 5i). Different heterocycles like pyrimidine, quinoline, pyrrole etc. were engaged in this study.^[68]^

**Scheme 6:**
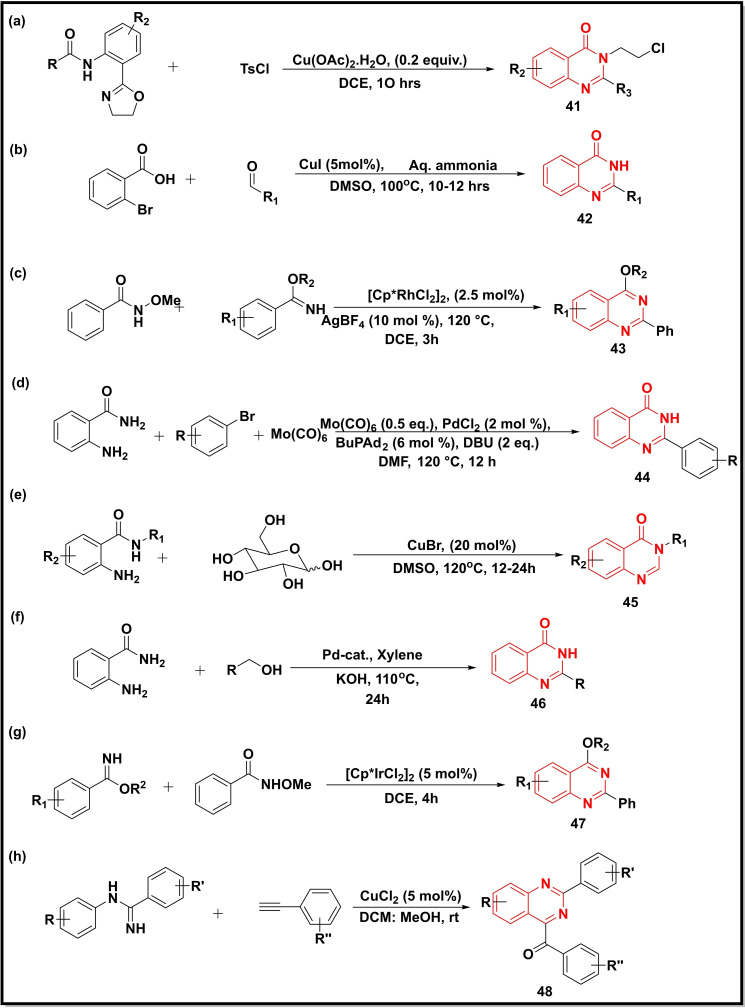
Synthesis of quinazoline‐4‐ones/quinazolines from amide‐oxazolines, arylamines, benzaldehydes, benzimidates, and amidines.

The synthesis of substituted quinazoline‐4‐ones (**41**) was accomplished through the cyclization of amide‐oxazolines with Tosyl chloride (TsCl) in the presence of DMAD and Cu(OAc)_2_ (Scheme 6a). However, this methodology suffered from substrate tolerance with different functional groups.^[69]^ A series of C2‐aryl/ heteroaryl quinazolines (**42**) were synthesized via oxidative coupling of 2‐bromobenzoic acid with aldehydes in the presence of CuI and aq. Ammonia (Scheme 6b). This protocol facilitated the double amination of aryl halides and acids into corresponding amines and amides present in the same substrate.^[70]^ The rhodium(III)‐catalyst was utilized for the activation C−H bond and tandem coupling for the preparation of quinazolines (**43**) using *N*‐alkoxyamide and benzimidates as starting materials where the former acts as an amidating reagent (Scheme 6c).^[71]^ Another methodology involved the Pd‐catalysed synthesis of 2‐substituted quinazoline‐4‐ones (**44**) *via* coupling reaction between 2‐aminobenzonitrile/2‐aminobenzamide, aryl bromides and Mo(CO)_6_ (Scheme 6d)_._ The Mo(CO)_6_ acted as a Carbonyl source in this reaction.^[72]^ Another research group prepared 2‐substituted quinazoline‐4‐ones (**45**) using Copper metal as a catalyst and glucose and 2‐amino‐*N*‐phenylbenzamide as starting materials (Scheme 6e). This methodology was atom‐economical and required benign conditions using *D*‐glucose as a multi‐C1 synthon.^[73]^ A Pd catalyst, [Pd(L)Cl(PPh_3_)] (L=dimethylamino benzoylhydrazone ligands) complex designed by Balaji *et al*. also displayed excellent activity for the synthesis of 2‐substituted quinazoline‐4‐ones (**46**) via dehydrogenative coupling between 2‐aminobenzamide and benzyl alcohols (Scheme 6f).^[74]^ The utilization of Ir‐catalyst [Cp*IrCl_2_]_2_ in the preparation of quinazolines (**47**) was demonstrated by treating benzamide esters with *N*‐methoxybenzamide in the presence of AgSbF_6_ (Scheme 6g). The functional group substituted at the *o‐*position reacted more rapidly than their *meta‐* and *para‐*counterparts. The synthetic strategy was found to be mild and tolerant to different functional groups.^[75]^ Charpe and co‐workers prepared quinazolines (**48**) photocatalytically via C_sp_^2^−H coupling of amidines with terminal alkynes, using CuCl_2_ as a photocatalyst and Oxygen as a green oxidant (Scheme 6h).^[76]^

**Scheme 7:**
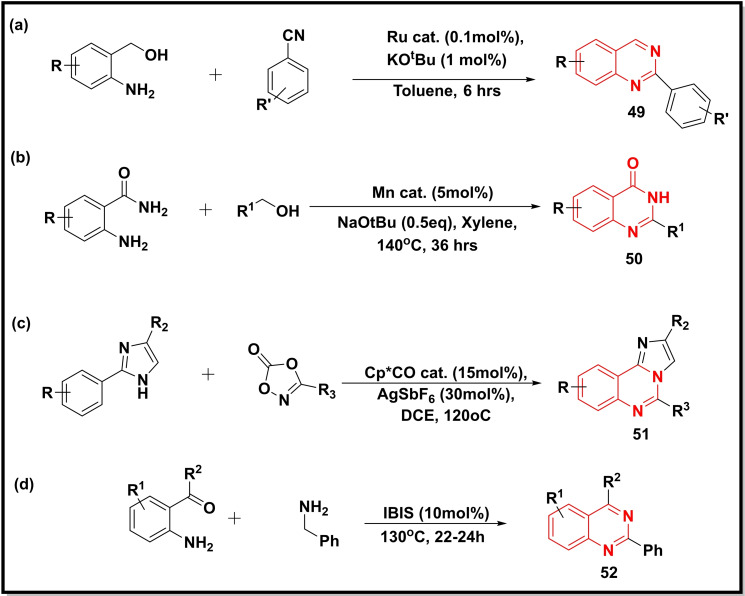
Synthesis of quinazoline‐4‐ones/quinazolines from substituted anilines and imidazoles.

Another study developed an effective Ruthenium pincer complex for the synthesis of quinazolines (**49**) via acceptorless dehydrogenative coupling (ADC) reaction of 2‐aminobenzyl alcohols with secondary alcohols (Scheme 7a). The catalyst achieved a high turnover number TON (290000) for 2‐phenylquinazoline using 1 mol % base loading and 0.0001 mol % loading of the catalyst.^[77]^ Pal and co‐workers performed the Mn‐catalysed condensation of anthranilamide with different primary alcohols (aromatic, aliphatic, and heteroaromatic) for preparing 2‐substituted quinazolin‐4‐ones (**50**) via ADC in moderate to good yield (50–84 %) (Scheme 7b).^[78]^ A CoIII‐ catalysed (Cp*CoIII) synthesis of imidazo[1,2‐c]quinazolines (**51**) via C−H coupling between 2‐aryl‐1H‐imidazoles and 1,4,2‐dioxazol‐5‐one using AgSbF_6_ as an additive was achieved (Scheme 7c). This methodology was claimed to be devoid of any hazardous side product.^[79]^ Martos *et al*. developed an environment‐benign iron (III)‐based catalyst and used it for condensation reaction between 2‐formyl/2‐acylanilines, and benzylamines for the synthesis of substituted quinazolines (**52**) in good to excellent yield (up to 93 %) (Scheme 7d).^[80]^

**Scheme 8:**
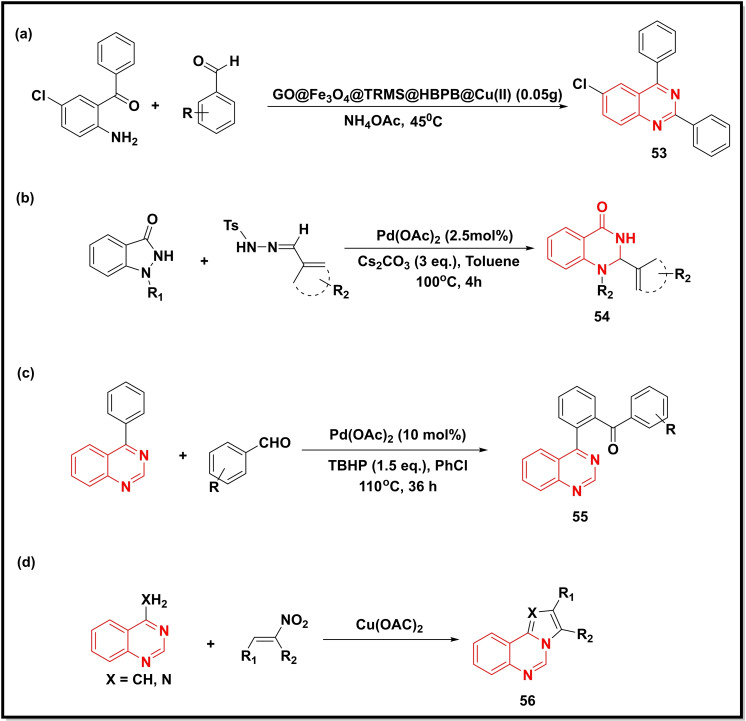
Synthesis of quinazoline/quinazoline‐4‐one analogues from substituted anilines, indoline and quinolines.

A novel recyclable nanocatalyst, (GO@Fe_3_O_4_@3‐chloropropyltrimethoxysilane@(Z)‐*N’*‐(2‐hydroxybenzylidene)‐4‐(pyridin‐4‐yl) benzohydrazide@Cu(II)) was employed in a multicomponent reaction of 5‐chloro‐2‐aminobenzophenone, aromatic benzaldehydes and ammonium acetate to prepared a new series of trisubstituted quinazolines (**53**) under solvent‐free conditions (Scheme 8a). The catalyst was highly efficient and recycled four times without any significant loss of the product yield.^[81]^ Chikkagundagal et al used Pd(OAc)_2_ to prepare substituted quinazolinones (**54**) using indazolones and tosyl hydrazones as precursors (Scheme 8b). The reaction initially involved the decomposition of tosyl hydrazones in the presence of base‐promoted palladium carbenoid, followed by nucleophilic attack of indazolones and finally intramolecular ring expansion through the N−N bond cleavage.^[82]^ The application of the same metal catalyst (Pd(OAc)_2_) in combination with tert‐butyl hydroperoxide (TBHP) was illustrated in the synthesis of quinazoline‐tagged aromatic ketones (**55**) by performing *ortho*‐aroylation of 4‐phenyl quinazoline using different aromatic aldehydes in chlorobenzene (Scheme 8c). The control experiments performed further suggested the participation of benzoyl radical pathway in these reactions.^[83]^ Following the same approach, Natalia Moreira and co‐workers performed the C−H functionalization of 4‐methylquinazolines (Scheme 8d) with β‐nitrostyrenes using copper acetate to afford the synthesis of new pyrrolo‐quinazolines and imidazo‐quinazolines (**56**).^[84]^

### 2.2 Non‐metal Catalysed Synthesis

**Scheme 9:**
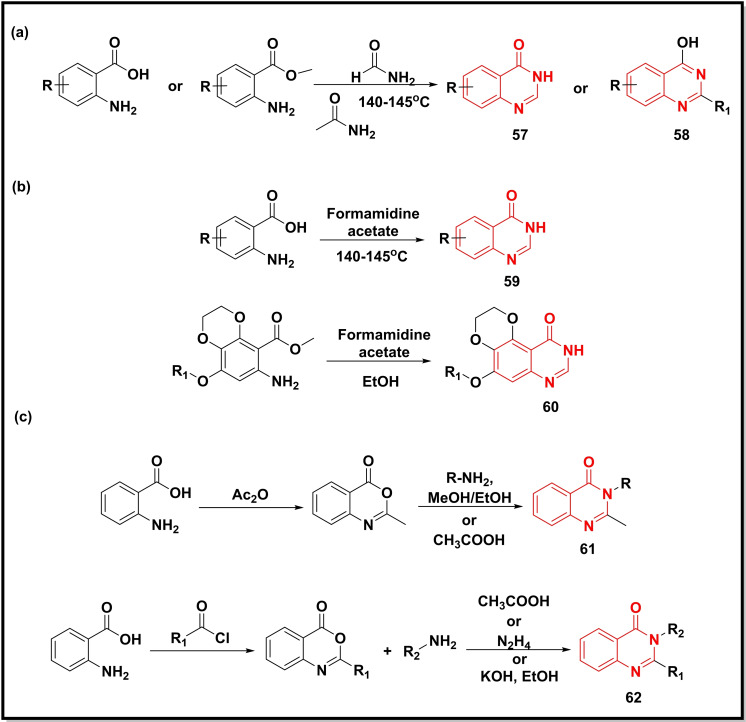
Synthesis of quinazolines/quinazoline‐4‐ones from substituted anilines, pyrazole amine, and different amidines.

The cyclisation of anthranilic acid or its ester with formamide or acetamide in an acidic medium afforded the quinazolines (**57**, **58**) either as a main product or as an intermediate in the reaction, in good yields (Scheme 9a).[^18^, ^22^, ^85^] In another study, quinazoline‐4‐one analogues (**59**, **60**) and other fused analogues were synthesised from substituted anthranilic acid and formamidine acetate in the presence of a catalyst. All the reactions were carried out under reflux conditions affording the desired quinazoline‐4‐ones in a shorter period with appreciable yields (85–93 %) (Scheme 9b).[^11^, ^86^, ^87^] The reaction of similar precursors, anthranilic acid with acetic anhydride or substituted acid chlorides resulted in 2‐substituted‐benzo[*d*][1,3]oxazin‐4‐one as intermediate which upon further condensation with different amines *viz*. hydrazine hydrate, urea, guanidine, hydrazides, thiourea, metformin and substituted anilines under acidic/basic conditions yielded the 2,3‐substituted quinazolin‐4‐ones (**61**, **62**) in high yields (80–92 %) (Scheme 9c).[^88^, ^89^, ^90^] Chalcone derivatives of dihydryonaphthalene after cyclisation with amino‐pyrazole resulted in heterobicyclic pyrazoloquinazolines.^[91]^

**Scheme 10:**
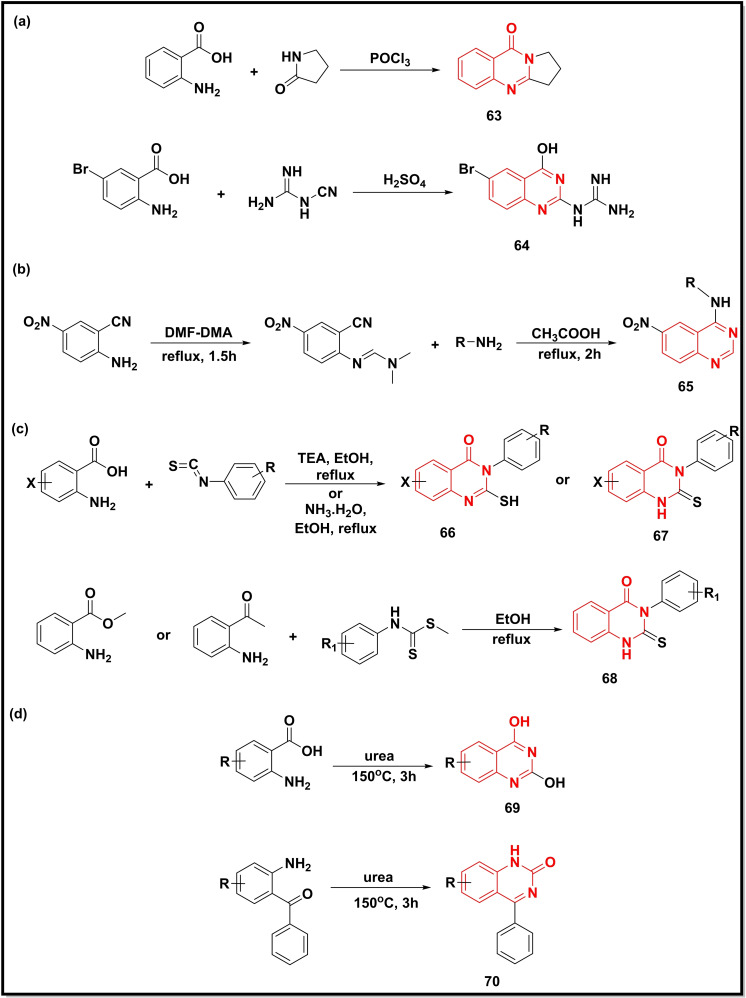
Synthesis of Synthesis of quinazolines/quinazoline‐4‐ones from substituted aryl amines.

The condensation of anthranilic acid with γ‐lactam or cyanoguanidine in the presence of a condensing agent (POCl_3_, H_2_SO_4_) furnished quinazoline analogues, deoxyvasicinone (**63**) and quinazolineguanidine (**64**) (Scheme 10a).^[92]^ Further, 2‐amino‐5‐nitrobenzonitrile after condensation with dimethylformamide (DMF)‐dimethyl acetal (DMA) produced (*E*)‐*N*′‐(2‐cyano‐4‐nitrophenyl)‐*N*, *N*‐dimethylformamide, which upon additional reaction with different aniline derivatives afforded 4‐substituted quinazolines (**65**) (Scheme 10b).^[93]^ Another study involved the cyclization of anthranilic acids with substituted isothiocyanates furnishing 2‐mercaptoquinazoline‐4‐one (**66**) or 2‐thiaoxoquinazoline‐4‐one (**67**) analogues under strong basic conditions.[^94^, ^95^] On the other hand, the reactions of anthranilates with substituted dithiocarbamic acids yielded 2‐thioquinazoline derivatives (**68**) (Scheme 10c).^[96]^ Both these methods were reported to be convenient routes for the access of sulfur‐containing quinazoline derivatives. Cyclisation of urea with anthranilic acid gives quinazoline‐2,4‐diol (**69**) at high temperatures with short time intervals in excellent yield (90.26 %).^[17]^ However, 2‐aminophenyl‐phenyl methanone, when reacted with urea, produces quinazoline (**70**) in a very low yield (7 %) (Scheme 10d).^[97]^

**Scheme 11:**
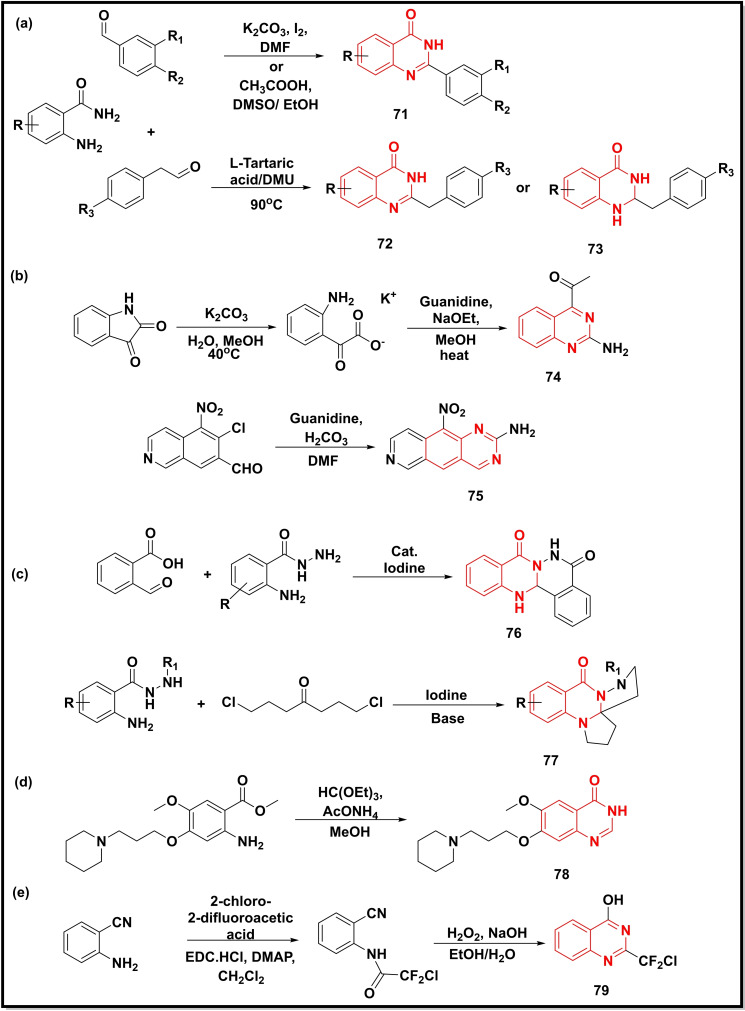
Synthesis of quinazoline‐4‐ones/quinazolines from substituted anilines, isatin and benzoic acid analogues.

The reaction of 2‐aminobenzamides (or anthranilamides) with substituted aromatic benzaldehydes afforded the 2‐substituted quinazoline‐4‐ones in acidic and basic media.[^19^, ^98^] Similarly, cyclization of the same anthranilamides with phenylacetaldehyde in the presence of tartaric acid and dimethylurea (DMU) produced the corresponding quinazoline‐4‐ones (**71–73**) in high yields (~80 %) (Scheme 11a).^[99]^ The commercially available isatin was hydrolysed to the amino acid potassium salt, followed by its cyclization with guanidine under basic conditions to yield 2‐aminoquinazoline (**74**) (Scheme 11b).^[100]^ In another study, the coupling reaction of guanidine salt with the quinoline intermediate led to the formation of a tricyclic quinazoline derivative (**75**) in 50 % yield (Scheme 11b).^[101]^ Iodine catalysed synthesis of fused quinazoline‐4‐one derivatives was carried out by two different research groups, where Jin *et al*. prepared phthalazino quinazoline‐4‐one (**76**) series starting from the 2‐formyl benzoic acid and 2‐aminobenzohydrazide,^[15]^ whilst Zhang *et al*. employed different substituted 2‐aminobenzohydrazides and 1,7‐dichloroheptan‐4‐one to generate tetracyclic pyrrolo‐quinazoline‐4‐one series (**77**) (Scheme 11c).^[13]^ Whereas, Methyl‐2‐amino‐5‐methoxy‐4‐(3‐(piperidin‐1‐yl)propoxy)benzoate was cyclised to afford a piperidine analogue of monocyclic quinazoline‐4‐one (**78**) by heating with triethyl orthoformate and ammonium acetate in alcohol (Scheme 11d).^[14]^ On the other hand, the synthesis of fluorinated‐quinazoline (**79**) initially involved the acetylation of 2‐aminobenzonitrile with the 2‐chloro‐2‐difluroacetic acid and a complexing reagent. Subsequently, the oxidative hydration of the fluorinated acylated intermediate with H_2_O_2_ followed by cyclisation in an alkaline medium afforded fluorinated quinazoline (Scheme 11e).^[102]^

**Scheme 12:**
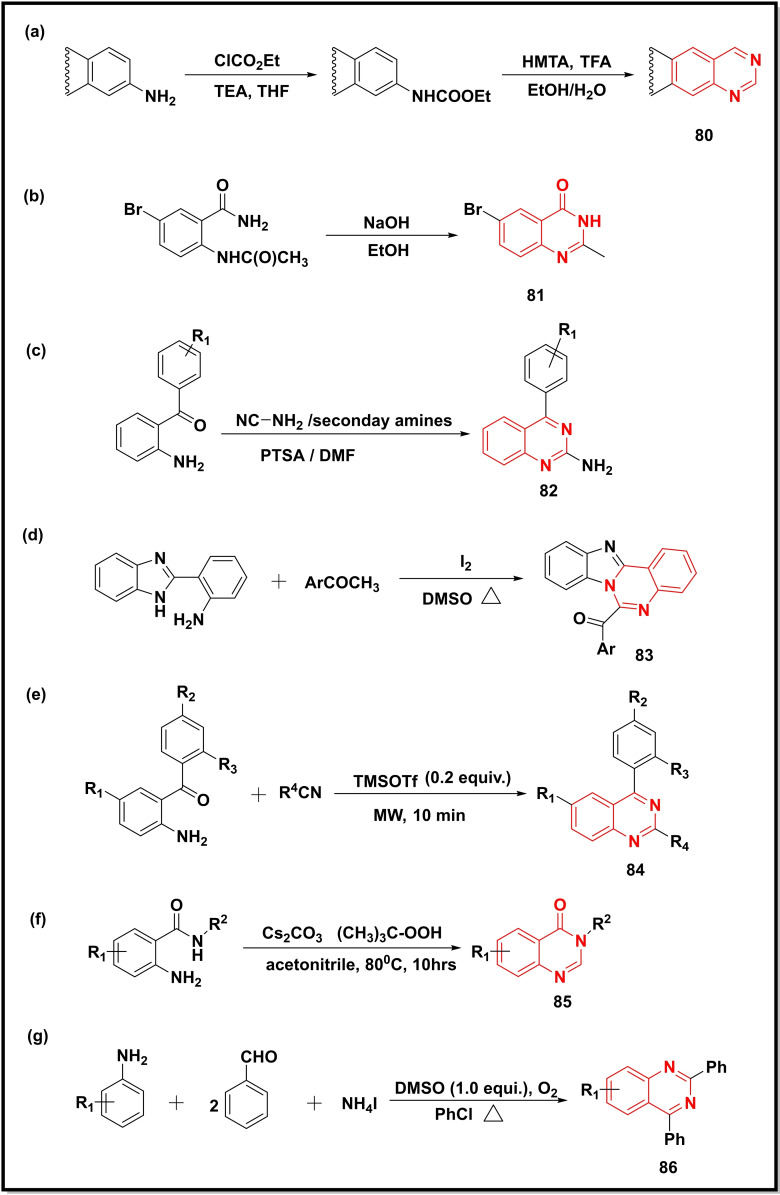
Synthesis of quinazolines/quinazoline‐4‐ones from substituted anilines and benzimidazole analogues.

In a similar study, carbamate‐protected anilines underwent condensation with hexamethylenetetramine in trifluoroacetic acid under microwave irradiation and then aromatization with potassium ferricyanide in hydroalcoholic potassium hydroxide affording quinazoline (**80**) (Scheme 12a).^[103]^ Further to this, a base‐promoted cyclization of acetamido‐5‐bromobenzamide in ethanol resulted in the quinazoline‐4‐one (**81**) (Scheme 12b).^[104]^

Pandya *et al*. developed a simple methodology for the synthesis of 2‐amino quinazolines (**82**) using both acidic and basic conditions. The protocol was reported to be scalable and tolerant to different functional groups (Scheme 12c).^[105]^ Ambethkar and co‐workers employed iodine iodine‐catalyzed cross‐coupling reaction between methyl ketones and 2‐(1Hbenzo[d]imidazol‐2‐yl)aniline to synthesize benzimidazo[1,2‐c]quinazolines (**83**) (Scheme 12d). The reaction offered better yields in DMSO when compared with other solvents [DMF, NMP (N‐methyl pyrrolidone), toluene, and CH_3_CN]. The aromatic ketones were reported to have more reactivity than hetero‐aromatic ketones.^[106]^ A microwave‐promoted synthesis of 2‐substituted quinazolinones (**84**) using 2‐aminophenyl carbonyl compounds and different aliphatic/aromatic nitriles as substrates was reported using TMSOTf as a Lewis catalyst (Scheme 12e).^[107]^ Another study utilized *tert*‐Butyl hydroperoxide (TBHP) for oxidative amination of the C(sp3)−H bond of 2‐amino benzamides to synthesize quinazolin‐4(3*H*)‐ones (**85**) (Scheme 12f). The reaction proceeded through a radical pathway. TBHP was observed to play the role of a methyl group in the metal‐free aerobic environment.^[108]^ Chen *et al*. performed a four‐component reaction between aniline, ammonium acetate (a nitrogen source), and aromatic benzaldehydes under metal‐free conditions to synthesise substituted quinazolines (**86**) in good yields (Scheme 12g). The utilisation of the C−H bond at the *ortho* position of aniline under aerobic conditions, good functional group tolerance, and broad substrate scope were highlights of this work.^[109]^

**Scheme 13:**
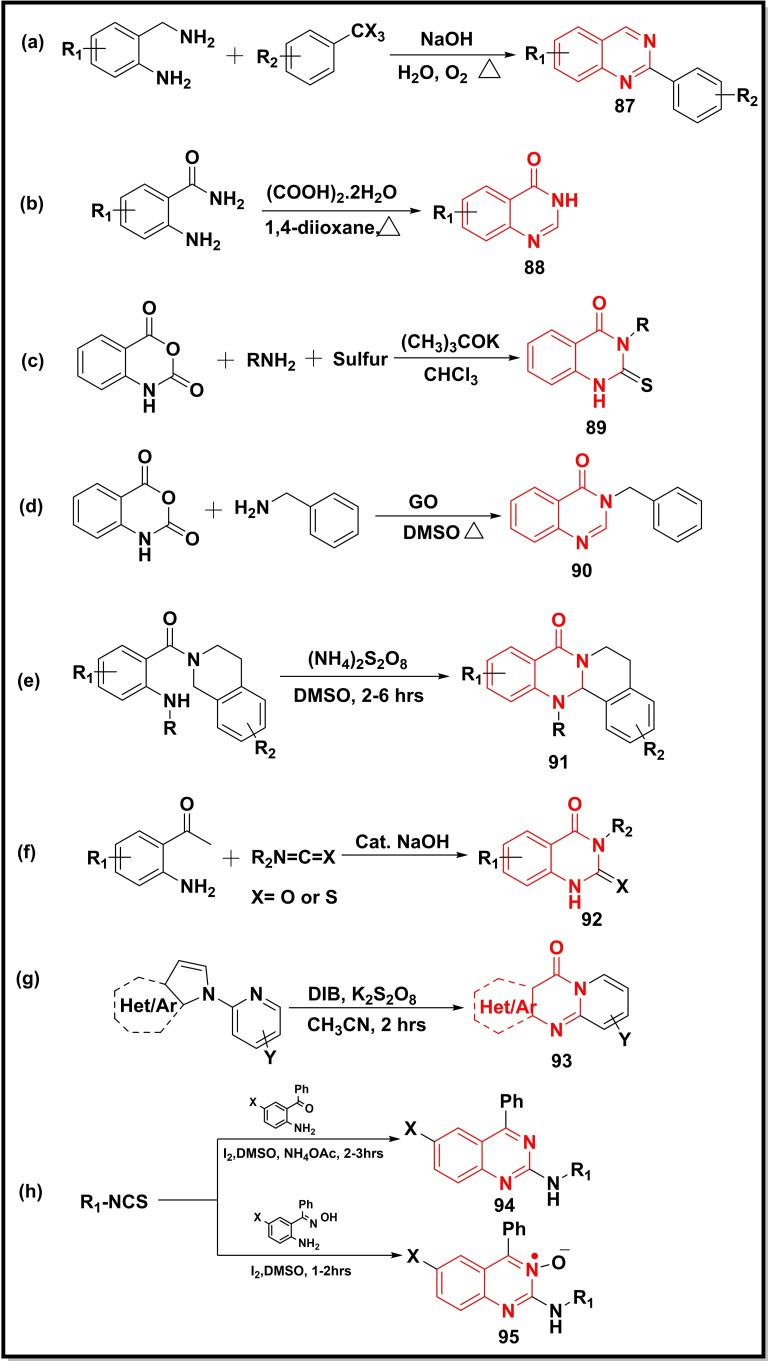
Synthesis of quinazoline‐4‐ones/quinazolines from substituted anilines, isatoic anhydride, fused pyrrolidine and isothiocyanates.

A base‐catalyzed synthesis of quinazoline (**87**) via condensation between 2‐aminobenzylamines and *α*,*α*,*α*‐trihalotoluene derivatives was disclosed (Scheme 13a). The methodology afforded multiple advantages like water as a solvent, molecular oxygen as an oxidant, readily available substrates, and cheap sodium hydroxide.^[110]^ The dual function of oxalic acid as a dehydrating agent and carbon source (in situ) was demonstrated in the preparation of quinazolinones (**88**) using 2‐amino benzamides as a precursor (Scheme 13b).^[111]^ Bahadorikhalili *et al*. developed a novel methodology for the preparation of 3‐substituted 2‐thioxo‐2,3‐dihydroquinazolin‐4(1*H*)‐ones (**89**) using isotonic anhydride, amines, sulfur, and t‐BuOK as substrates (Scheme 13c). The mechanistic pathway revealed the participation of carbene as an intermediate under basic conditions.^[112]^ The use of GO (graphene oxide) in the synthesis of quinazolinone (**90**) using isotonic anhydride and benzylamine was demonstrated in DMSO which served as a solvent as well as a carbon source in this reaction (Scheme 13d).^[113]^ Along the same line, the ammonium persulphate (NH_4_)_2_S_2_O_8_ triggered the synthesis of polycyclic quinazoline (**91**) (Scheme 13e) via intramolecular cyclization of 2‐aminobenzamide. The synthetic protocol was further employed to synthesise two natural products, rutaecarpine and tryptanthrin. The mechanistic study revealed the intermediacy of radical species in the reaction.^[114]^ The sodium hydroxide catalyzed synthesis of 3‐substituted 4‐methylene‐3,4‐dihydroquinazoline‐2(1H)‐thiones/ones (**92**) via intermolecular cyclization between 2‐aminoacetophenone and isothiocyanate was reported (Scheme 13f).^[12]^ The combination of potassium persulphate K_2_S_2_O_8_ and DIB [(diacetoxyiodo)benzene] was used to synthesize fused quinazolinones (**93**) from N‐pyridyl indoles (Scheme 13g). These protocols have shown significant properties like broad substrate scope, metal‐free condition, short reaction time, and operational simplicity.^[115]^ Two different series of disubstituted quinazoline‐2‐amines were obtained using iodine as a catalyst (Scheme 13h). The reaction between isothiocyanates and 2‐amino benzophenone in DMSO and molecular iodine afforded the first series (**94**), while the second series **95** was prepared using (2‐aminophenyl)(phenyl)methanone oxime as a precursor using the same reaction conditions.^[116]^

**Scheme 14:**
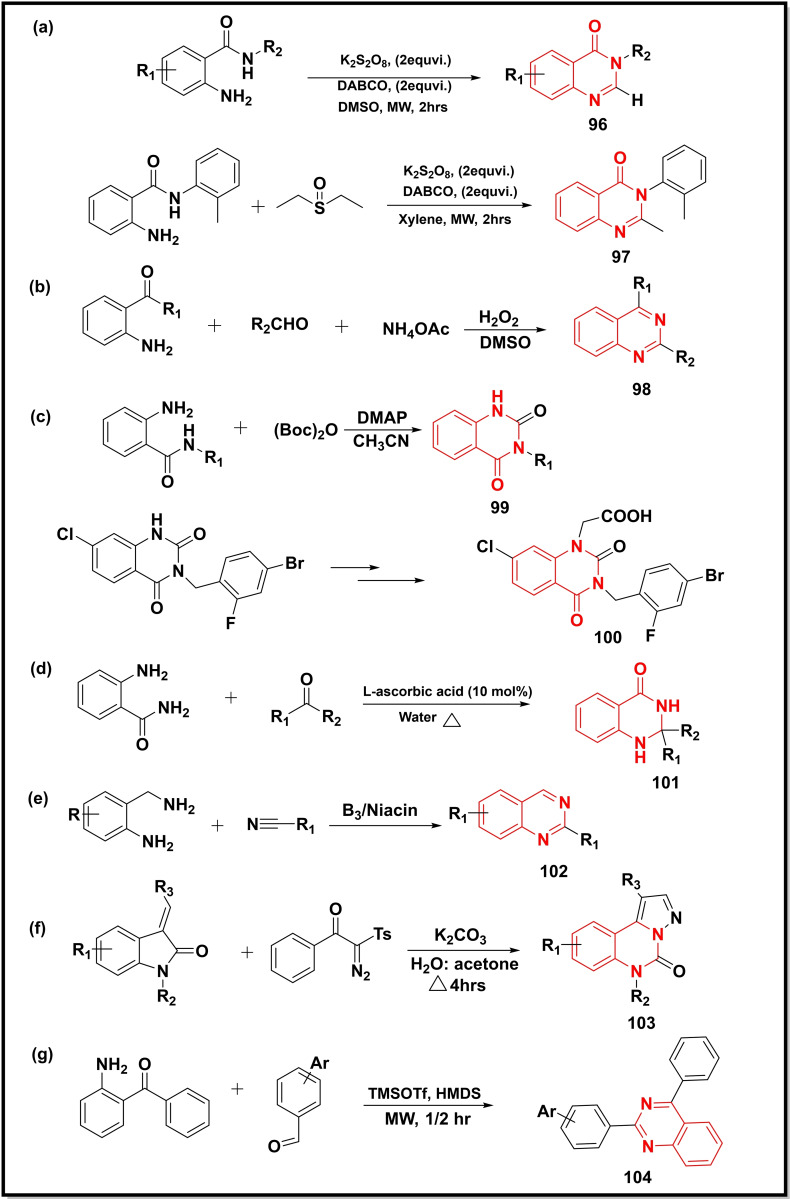
Synthesis of quinazolines/quinazoline‐4‐ones from substituted anilines and isatin analogues.

Another study utilized K_2_S_2_O_8_ for the preparation of quinazolinones (**96**) from 2‐aminobenzamides using DMSO as a methane source (Scheme 14a). The significance of the protocol was further demonstrated by synthesizing a known drug, Methaqualone (**97**).^[117]^ A one‐pot three‐component reaction, in another study, employing 2‐amino aryl ketones, ammonium acetate, and aldehydes led to the generation of 2,4‐substituted quinazolines (**98**) (Scheme 14b). The protocol was claimed to yield high product yield, readily available hydrogen peroxide as oxidant, broad substrate scope, metal‐free catalyst, and mild reaction conditions.^[118]^ The 4‐dimethylamino pyridine catalyzed one‐pot synthesis of quinazoline‐2,4‐diones (**99**) via cyclization of 2‐aminobenzamide in the presence of (Boc)_2_O was achieved, where the latter donated a carbonyl moiety to the quinazoline core at the 3‐position (Scheme 14c). The use of microwave energy offered the desired product in a shorter time. The p‐methoxybenzyl (PMB) utilized for activation of cyclization smoothly activated cyclization at room temperature instead of the MW energy. Zenarestat (an aldose reductase inhibitor) **100** was also synthesized successfully in this study using the same protocol in good yield (70 %).^[119]^ Another study utilized L‐ascorbic acid as a metal‐free catalyst for the synthesis of quinazolin‐4(3*H*)‐ones (**101**) via cyclization of 2‐aminobenzamide with aldehydes as well as with ketones in water using oxone as oxidant, in good to excellent yield (70–92 %) (Scheme 14d).^[120]^ Gujjarappa *et al*. used niacin (vitamin‐B_3_) to cyclize 2‐aminobenzylamines with nitriles for the preparation of 2‐substituted quinazolines (**102**) in good yields (up to 91 %) (Scheme 14e). The protocol was reported to be tolerant towards a wide range of functional groups.^[121]^ A base‐promoted 1,3 dipolar cycloaddition between 3‐ylideneoxindoles and tosyldiazomethane in H_2_O resulted in pyrazolo‐[1,5‐c]quinazolines (**103**) in moderate to good yield (60–90 %) (Scheme 14f). The use of green solvent, ring expansion, and regioselectivity were reported to be the main highlights of the work.^[122]^ The combination of MW energy and TMSOTf and HMDS (hexamethyldisilazane) catalysts led to the preparation of quinazolines (**104**) via coupling between 2‐amino benzophenone and different benzaldehydes (Scheme 14g). The HMDS played the role of an *in‐situ* nitrogen source in the reaction.^[123]^

### 2.3 Miscellaneous Reactions

**Scheme 15:**
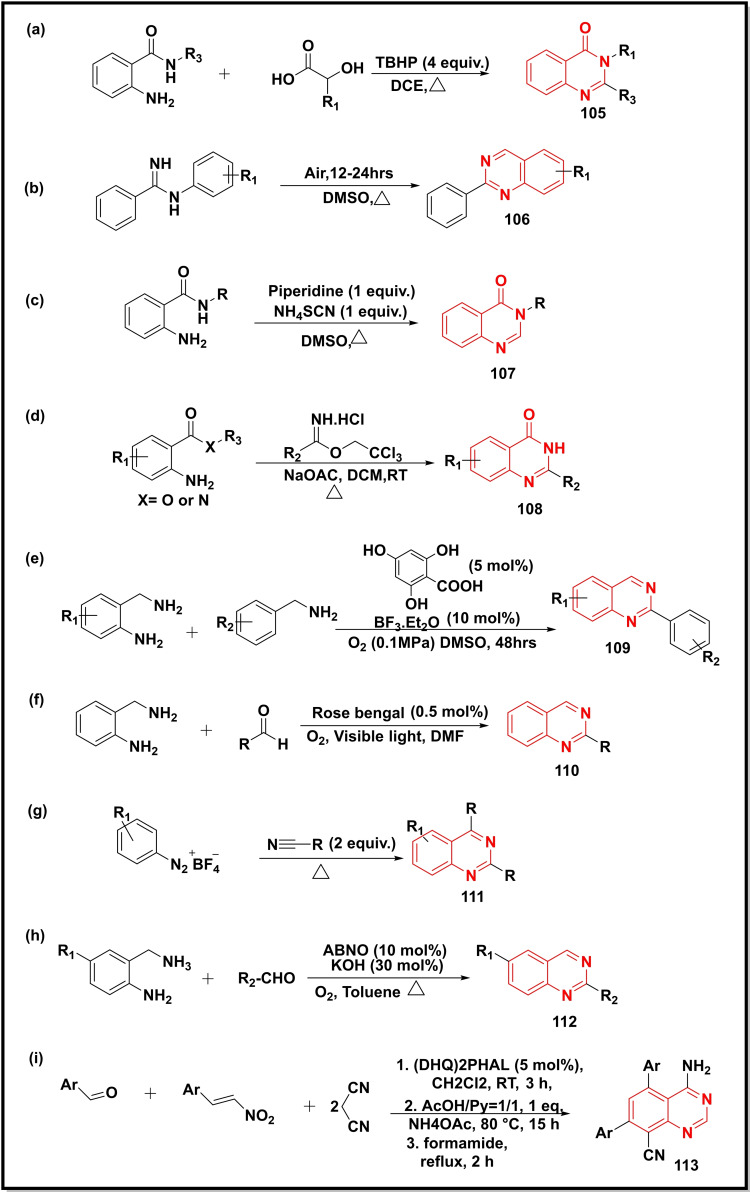
Synthesis of quinazolines/quinazoline‐4‐ones from substituted anilines, amidines, benzene diazonium salts and nitroalkenes.

The role of TBHP (tert‐butyl hydroperoxide) in the synthesis of quinazoline‐4‐ones (**105**) via condensation between 2‐amino benzamides and α‐hydroxy acids was demonstrated (Scheme 15a). The α‐hydroxy acids were used as a carbonyl source to produce *in‐situ* aldehydes. The application of the protocol was also demonstrated by synthesizing a bioactive compound, methaqualone (R_1_=*o*‐tolyl, R_3_=CH_3_) in good yield (63 %).^[124]^ Synthesis of 2‐substituted quinazolines (**106**) via intramolecular cyclization of benzimidamide in DMSO was reported in good yields (up to 92 %) at 165 °C (Scheme 15b). The mechanistic studies revealed the role of DMSO as a carbon source.^[125]^ The NH_4_SCN‐promoted synthesis of quinazolinones (**107**) by the cyclisation reaction of 2‐aminobenzamide in the presence of piperidine and DMSO was reported (Scheme 15c). The DMSO played the role of solvent as well as carbon source.^[126]^ In another study, sodium acetate was utilized in the preparation of 2‐substituted quinazolinones (**108**) from 2‐aminobenzamides/methyl 2‐aminobenzoates and 2,2,2‐trichloroethyl imidates hydrochloride in DCM (Scheme 15d).^[127]^ The use of salicylic acid as a catalyst in the synthesis of 2‐substituted quinazolines (**109**) via one‐pot condensation between 2‐aminobenzylamines and substituted benzylamines in DMSO was demonstrated (Scheme 15e). The BF_3_⋅Et_2_O (10 mol %) was used as a Lewis acid for oxidative intramolecular cyclization. The methodology was reported to achieve excellent green protocols like RME (73 %), tremendous atom economy, and an E‐factor (2.7).^[128]^ The singlet oxygen‐driven synthesis of quinazolines (**110**) was accomplished by cyclization of 2‐amino benzylamine and aldehydes in the presence of rose bengal under visible light irradiations in moderate to good yield (45–92 %) (Scheme 15f).^[129]^ The [2+2+2] cyclization of aryldiazonium salts with two equivalents of nitriles provided substituted quinazolines (**111**) in excellent yields (96–98 %) yield (Scheme 15g).^[130]^ The 9‐azabicyclo[3.3.1]nonan‐N‐oxyl (ABNO) and KOH also catalysed the coupling of 2‐aminobenzylamines with aldehydes using molecular oxygen. This protocol offered quinazolines (**112**) in a shorter reaction time as compared to visible light (Scheme 15h).^[131]^ Another study synthesized the 4,5,7,8‐substituted quinazolines (**113**) via a one‐pot reaction between aryl aldehydes, nitroalkanes, and malononitrile (Scheme 15i).^[132]^

**Scheme 16:**
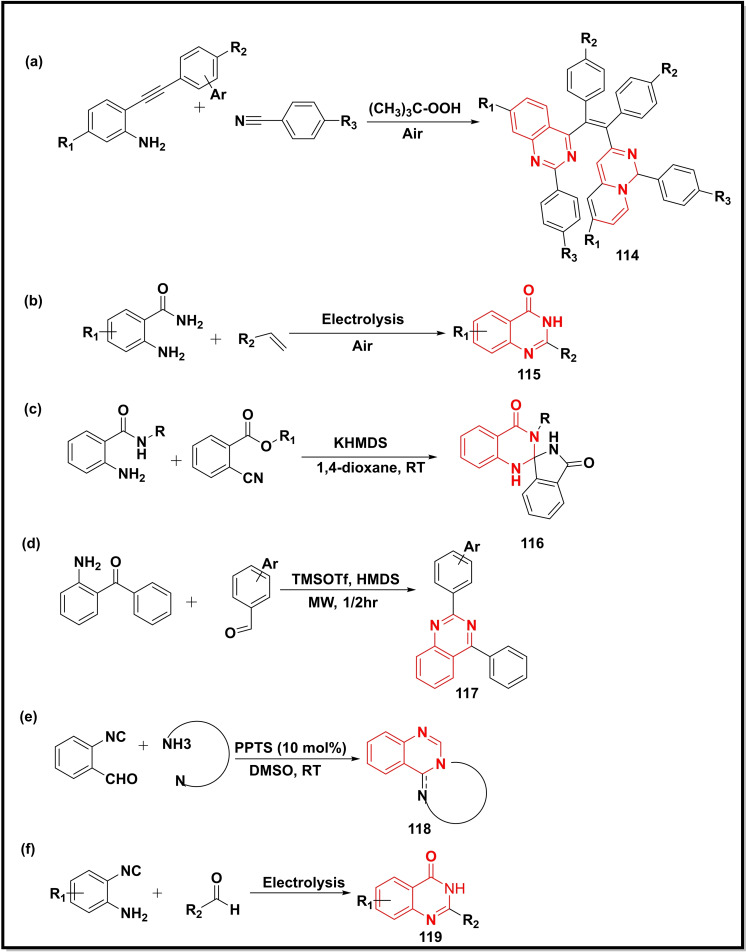
Synthesis of quinazolines/quinazoline‐4‐ones from substituted anilines, benzophenone, and aryl cyanide analogues.

Synthesis of π‐conjugated quinazoline‐substituted ethane derivatives (**114**) was achieved via cyclization of benzonitrile with 2‐ethynyl anilines using *t*‐BuOK as a base under aerobic conditions (Scheme 16a). The methodology provided high yields of the compounds with Z‐selectivity.^[133]^ Another study reported the synthesis of 2‐substituted quinazoline‐4‐ones (**115**) using electrolysis methods starting from 2‐aminobenzamides and alkenes with broad substrate scope and high yield (Scheme 16b).^[134]^ The KHMDS (potassiumhexamethylsilazide) was used in the synthesis of spiro‐isoindolinone dihydroquinazolinones (**116**) through cyclization of 2‐aminobenzamides and 2‐cyanomethyl benzoates (Scheme 16c).^[135]^ A combination of TMSOTf and HMDS (hexamethyldisilazane) was used for preparing quinazolines (**117**) (83–93 %) via coupling of 2‐aminobenzophenone with various benzaldehydes under microwave irradiations (Scheme 16d).^[123]^ Synthesis of fused quinazolines (**118**) was accomplished by cyclization of 2‐isocyanobenzaldehydes with various amines using PPTS (pyridinium *p*‐toluenesulfonate) as a catalyst. The DMSO was observed to be the best solvent as compared to other solvents (DMF, THF, DCE, MeCN, and 1,4‐dioxane) to carry out these reactions (Scheme 16e).^[136]^ Another study utilized electrochemical energy to cyclize *o*‐aminobenzonitriles in the presence of aldehydes, I_2,_ and KOH to form 2‐substituted quinazolin‐4‐ones (**119**) in an aqueous medium (Scheme 16f).^[137]^

**Scheme 17:**
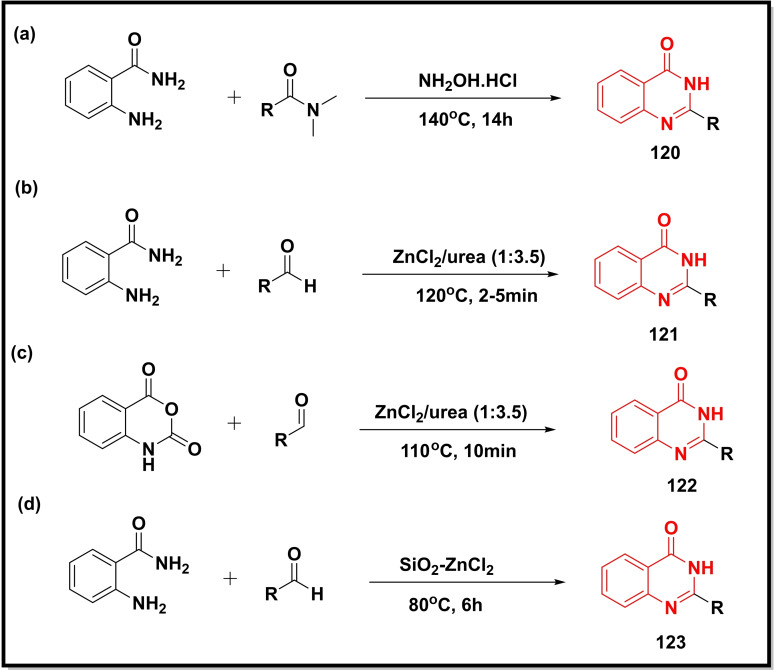
The synthesis of quinazolinones using transamidation strategy and deep eutectic solvent.

Shaik et al. reported the synthesis of quinazolinones (**120**) using a transamidation strategy whereby a one‐pot reaction between 2‐aminobenzamide and various amides led to the target compounds without employing any solvent (Scheme 17a).^[138]^ We also developed a green and sustainable methodology for the synthesis of quinazolin‐4‐ones (**121**) via condensation of 2‐aminobenzamide with various aldehydes using deep eutectic solvent (DES) (ZnCl_2_/urea) (Scheme 17b). DES played a dual role by acting as both a solvent as well a catalyst. The protocols provided remarkable advantages such as column‐free isolation, excellent yields, broad substrate scope, and reusability of the catalyst.^[139]^ In another study, we used the same DES to couple isatoic anhydride with aldehydes to synthesise 2‐substituted quinazolin‐4‐ones (**122**) (Scheme 17c). Here, the DES played a triple role as substrate (nitrogen source), solvent, and catalyst.^[140]^ Another study synthesized a catalyst (SiO_2_‐ZnCl_2_) to facilitate the condensation of 2‐aminobenzaminde with aldehydes for the synthesis of quinazolin‐4‐ones (**123**) (Scheme 17d).^[31]^

## 3 Biological Importance of Quinazolines/quinazolin‐4‐ones

Quinazoline derivatives have shown activity in a vast array of biological assays including antimicrobial, anticancer, antitubercular, antimalarial, analgesic, anti‐inflammatory, anticonvulsant, anti‐epileptic, and anti‐HIV, and have been reviewed extensively in the last three years.[^8^, ^9^, ^10^] The quinazoline scaffold can be functionalised at a variety of positions, however, the most common positions explored are N‐1, C‐2, N‐3, C‐4, and C‐6. In the following section, the anticancer and antimicrobial activities of some representative quinazoline//quinazoline‐4‐one derivatives are summarized.

### 3.1 Anticancer Activity

**Scheme 18:**
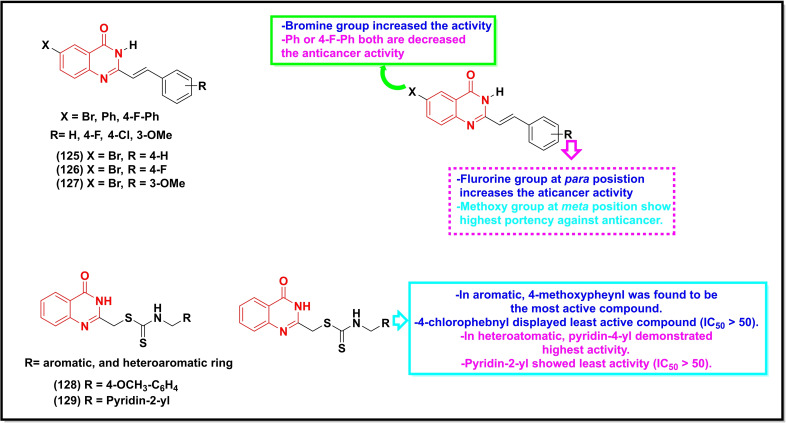
Showing general structures of tested quinazolin‐4‐ones and the potent anticancer compounds with their summarized SAR.

**Scheme 19:**
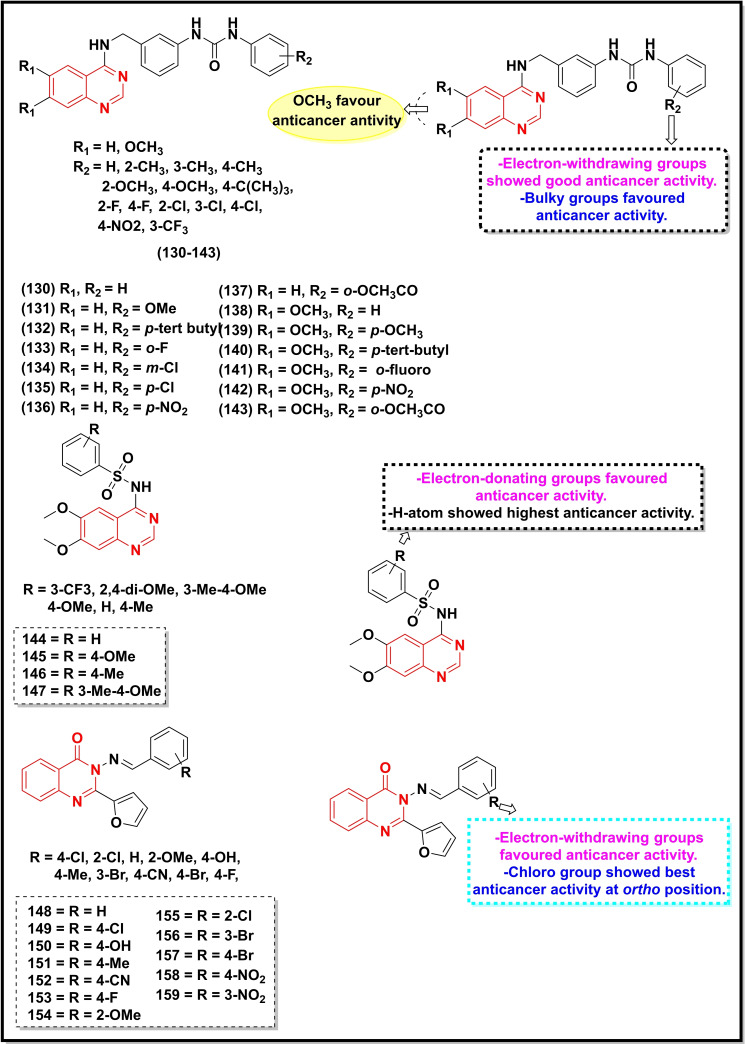
Illustration of potent anticancer quinazoline/quinazolin‐4‐one derivatives with their summarized SAR.

**Scheme 20:**
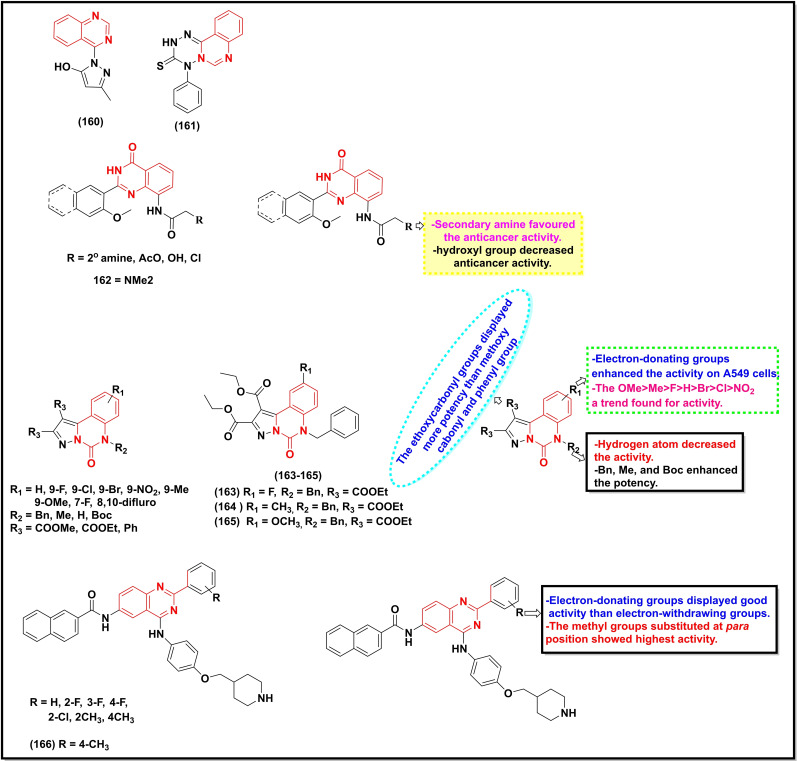
Showing general structures of tested quinazolinones and the potent anticancer compounds along with their summarized SAR.

**Scheme 21:**
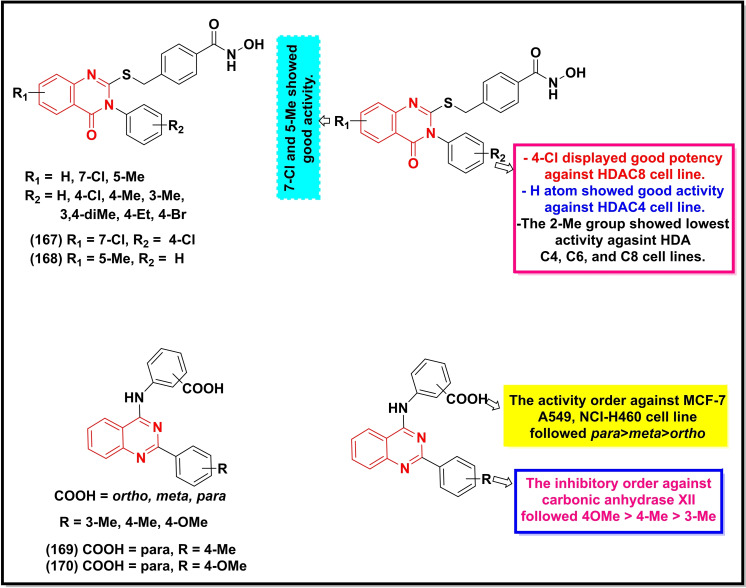
Showing general structures of tested quinazolines/quinazoline‐4‐ones and the potent anticancer compounds along with their summarized SAR.

A summary of the potential anticancer activity of some selected quinazoline/quinazoline‐4‐one derivatives (Scheme 18, 19, 20, 21) against 18 cancer cell lines with IC_50_/ or GI_50_ values <30 μM (or μg/ml in some cases) is reported in Table 1. Predominantly, these quinazolines were substituted at C‐2, C‐4, and C‐6.

**Table 1:** Anticancer activity of quinazoline/quinazoline‐4‐one analogues (IC_50_ in μM).

No.	TK10	UACC‐62		MCF‐7	MGC‐803	HepG2	A549	HT‐29	A375	A431	A54	HeLa	HCT‐116	Capan‐1	NCI‐H460	HL‐60	K‐562	Z‐138	DND‐41
125	7.72	1.98		2.12	–	–	–	–	–	–	–	–	–	–	–	–	–	–	–
126	2.42	1.03		3.47	–	–	–	–	–	–	–	–	–	–	–	–	–	–	–
127	1.12	0.62		1.80	–	–	–	–	–	–	–	–	–	–	–	–	–	–	–
128	–	–		–	–	0.5	–	–	–	–	–			–	–	–	–	–	–
129	–	–		10.86	–	–	7.27	5.53	–	–	–	12.19	22.72	–	–	–	–	–	–
130	–	–		–	13.24	–	–	–	–	–	–	–	–	–	–	–	–	–	–
131	–	–		–	–	17.45	–	–	–	–	–	–	–	–	–	–	–	–	–
132	–	–		–	7.47	16.95	–	–	–	–	–	–	–	–	–	–	–	–	–
133	–	–		–	–	17.47	–	–	–	–	–	–	–	–	–	–	–	–	–
134	–	–		–	19.22	–	–	–	–	–	–	–	–	–	–	–	–	–	–
135	–	–		–	26.38	–	–	–	–	–	–	–	–	–	–	–	–	–	–
136	–	–		–	9.42	–	–	–	–	–	–	–	–	–	–	–	–	–	–
137	–	–		–	9.63	–	–	–	–	–	–	–	–	–	–	–	–	–	–
138	–	–		–	10.02	19.72	–	–	–	–	–	–	–	–	–	–	–	–	–
139	–	–		–	12.28	–	–	–	–	–	–	–	–	–	–	–	–	–	–
140	–	–		–	6.79	6.85	3.21	–	–	–	–	–	–	–	–	–	–	–	–
141	–	–		–	10.32	–	–	–	–	–	–	–	–	–	–	–	–	–	–
142	–	–		–	8.12	18.84	–	–	–	–	–	–	–	–	–	–	–	–	–
143	–	–		–	8.98	–	–	–	–	–	–	–	–	–	–	–	–	–	–
144	–	–		6.06	–	–	–	–	–	–	–	–	–	–	–	–	–	–	–
145	–	–		8.25	–	–	–	–	–	–	–	–	–	–	–	–	–	–	–
146	–	–		33.54	–	–	–	–	–	–	–	–	–	–	–	–	–	–	–
147	–	–		39.47	–	–	–	–	–	–	–	–	–	–	–	–	–	–	–
148	–	–		–	–	–	–	–	–	–	–	–	–	–	–	50.34	40.21	–	–
149	–	–		–	–	–	–	–	–	–	–	–	–	–	–	28.38	10.38	–	–
150	–	–		–	–	–	–	–	–	–	–	–	–	–	–	32.76	34.89	–	–
151	–	–		–	–	–	–	–	–	–	–	–	–	–	–	24.89	62.14	–	–
152	–	–		–	–	–	–	–	–	–	–	–	–	–	–	32.23	64.36	–	–
153	–	–		–	–	–	–	–	–	–	–	–	–	–	–	32.38	60.45	–	–
154	–	–		–	–	–	–	–	–	–	–	–	–	–	–	32.38	37.22	–	–
155	–	–		–	–	–	–	–	–	–	–	–	–	–	–	38.98	48.82	–	–
156	–	–		–	–	–	–	–	–	–	–	–	–	–	–	12.24	25.85	–	–
157	–	–		–	–	–	–	–	–	–	–	–	–	–	–	4.38	27.23	–	–
158	–	–		–	–	–	–	–	–	–	–	–	–	–	–	13.63	62.59	–	–
159	–	–		–	–	–	–	–	–	–	–	–	–	–	–	25.85	26.39	–	–
160	–	–		16.40*		28.90*	–	–	–	–	–	–	–	–	–	–	–	–	–
161	–	–		20.50*		23.10*	–	–	–	–	–	–	–	–	–	–	–	–	–
162	–	–		–	–	–	–	–	–	–	–	–	2.2	1.8	1.4	10.6	12.7	2.6	5.5
163	–	–		–	–	–	17.0	–	–	–	–	–	–	–	–	–	–	–	–
164	–	–		–	–	–	14.2	–	–	–	–	–	–	–	–	–	–	–	–
165	–	–		–	–	–	18.1	–	–	–	–	–	–	–	–	–	–	–	–
166	–	–			1.95	–	–	–	–	–	–	–	–	–	–	–	–	–	–
167	–	–		24.31	–	–	–	–	–	–	–	–	17.62	–	–	–	–	–	–
168	–	–		13.65	–	–	–	–	–	–	–	–	12.38	–	–	–	–	–	–
169	20.8^#^	30^#^		2.98^#^	–	–	12.1^#^	3.37^#^	–	–	–	–	–	–	2.26^#^	5.59^#^	4.16^#^	–	–
170	29.3^#^	–		9.4^#^	–	–	8.31^#^	17.9^#^	–	–	–	–	10.1^#^	–	–	8.5^#^	7.05^#^	–	–

Key: TK10 (Human renal); UACC62 (Melanoma); MCF7 (Breast); MGC‐803 (gastric carcinoma); HepG2 (liver cancer); A549 (adenocarcinoma); HT‐29 (colorectal adenocarcinoma); A375 (human melanoma); A431 (epidermoid carcinoma); A54, HCT‐116 (colon cancer), HeLa (Cervical carcinoma). Capan‐1 (Pancreatic Adenocarcinoma); NCI‐H460 (Lung Carcinoma); HL‐60 (Acute Myeloid Leukemia); K‐562 (Chronic Myeloid Leukemia); Z‐138 (Non‐ Hodgkin Lymphoma); DND‐41 (Acute Lymphoblastic Leukemia); *activity in μg/ml; ^#^activity in growth inhibition (GI_50_)

Compounds **125–127** contained a substituted styryl group at C‐2. These compounds were active against Human renal (TK10) and Melanoma and Breast cancer (MCF‐7) with IC_50_ values between 0.62–7.72 μM.^[104]^ Ding and co‐workers synthesized a series of quinazolin‐4‐ones bearing dithiocarbamate moiety with various aromatic and heterocyclic groups at the C‐2 position and tested them against five cancer cell lines viz. A549, MCF‐7, HeLa, HT29 and HCT‐116. The most active compounds (**128** and **129**) of the series displayed noticeable activity against the tested cell lines with IC_50_ values ranging between 5.53 μM to 22.72 μM (Table 1).^[88]^

Compounds **130–143** containing aminophenyl‐phenylurea groups at C‐4 showed IC_50_ values between 3.21–19.72 μM against MGC‐803, HepG2, and A‐549 cell lines.^[85]^ Compounds **144–147** carrying sulphonamide group at C‐4 showed good activity with IC_50_ values ranging between 9.23 μM to 19.05 μM.^[141]^ Similarly, compounds **148–159** bearing Schiff base at C‐3 demonstrated good anticancer activity within a range of 2.3 μM to 173.2 μM against the HepG2 cell line.^[142]^

The placement of substituted pyrazole ring to the quinazoline at C‐4 (**160**) afforded good activity against MCF‐7 (IC_50_=16.40 μg/ml) and HepG2 (IC_50_=28.90 μg/ml), while its tetrazinoquinazoline counterpart **161** showed relatively less activity (IC_50_=20.50 μg/ml) against the former cancer cell line and improved activity against the latter (IC_50_=23.10 μg/ml).^[143]^ The positioning of the *ortho*‐methoxyphenyl group at C‐2 and a basic moiety at C‐8 resulted in even a highly potent compound **162** against a range of cancer lines (HCT‐116, Capan‐1, NCI‐H460, HL‐60, K‐562, Z‐138, and DND‐41) with IC_50_ values ranging between 1.4 μM to 127 μM.^[144]^ The testing of pyrazolo‐[1,5‐c]quinazolinones (**163–165**) disclosed three moderately active compounds against the A‐549 cell line with the most active compound displaying an IC_50_ value of 14.2 μM. However, only compound **165** was found to be active (IC_50_=26.2 μM) against the triple‐negative breast cancer cell line (MDA‐MB‐231) in this study.^[145]^ A recently reported 2,4,6‐trisubstituted quinazoline analogue (**166**) exhibited potent activity (IC_50_=~2 μM) against the gastric cancer line (MGC‐803) and human esophageal epithelial cancer cell line (Eca‐109), and induced dose‐dependent apoptosis and cell cycle arrest in S phase in the former cancer cell line.^[146]^

The *in vitro* testing of a series of quinazoline‐4‐one derivatives prepared by Yogesh *et al*. also revealed two potent compounds, **167** and **168** with IC_50_ values of 140± 20 and 400± 140 nM respectively against HDAC8 and HDAC4 cell lines.^[147]^ Two 2,4‐disubstituted quinazoline derivatives (**169**–**170**) also when exploited against a panel of 59 cancer cell lines exerted potent activity (IC_50_ <10 mM) for MCF‐7, A549, NCI‐H460, and K‐562 including strong inhibition of carbonic anhydrase XII (IC_50_=0.48 mM).^[148]^

### 3.2 Antimicrobial Activity

**Scheme 22:**
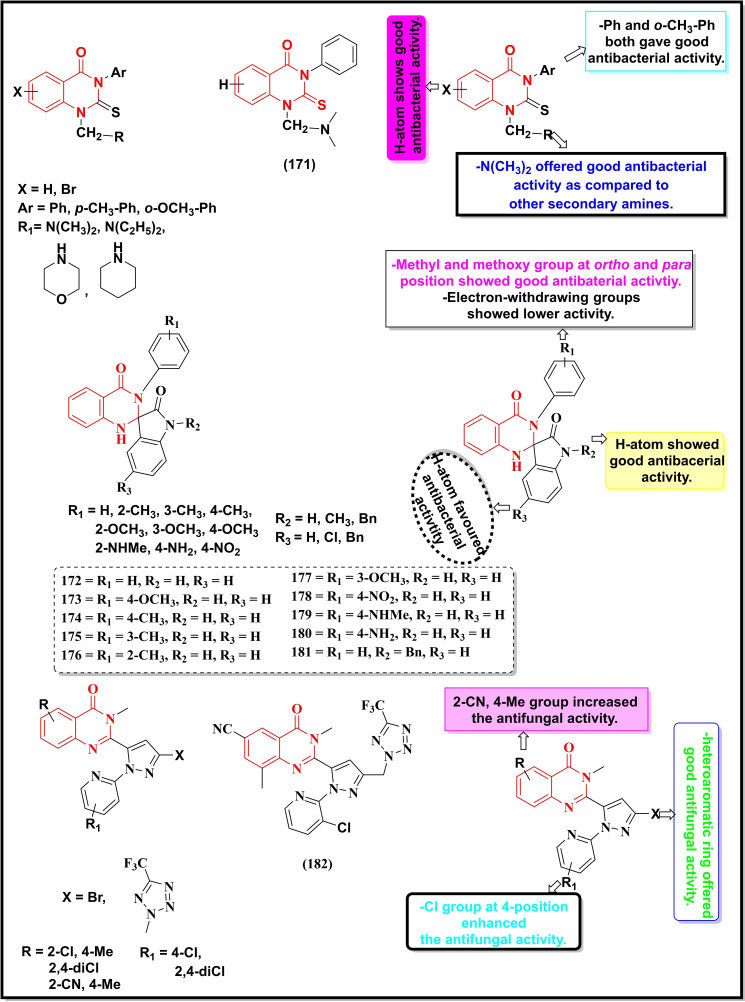
Showing general structures of tested quinazolin‐4‐ones and the potent antimicrobial compounds with their summarized SAR.

**Scheme 23:**
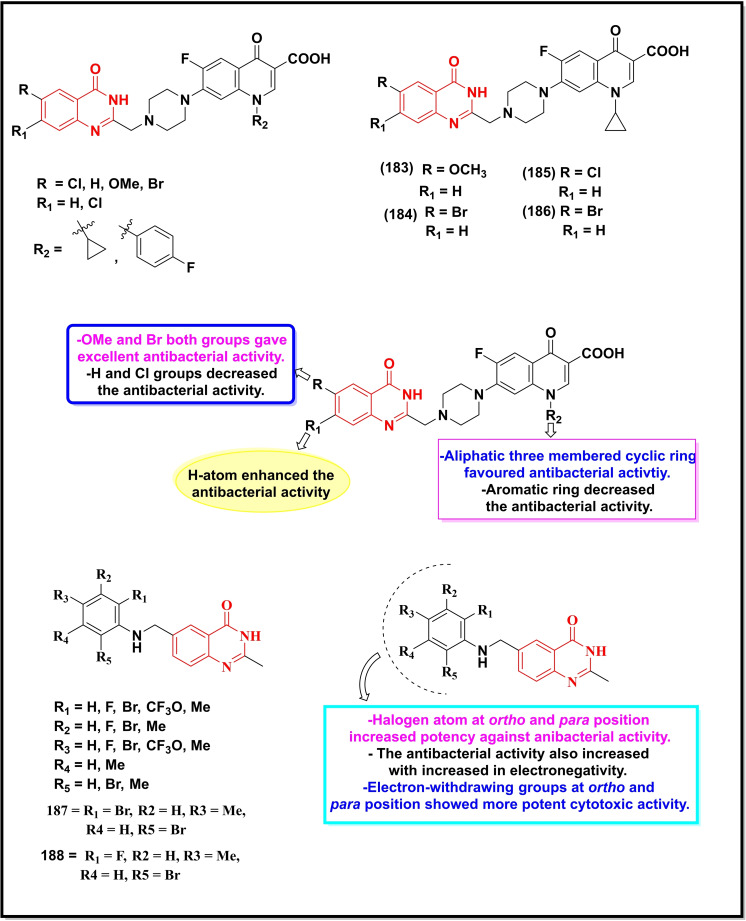
Showing general structures of tested quinazolin‐4‐ones and the potent antimicrobial compounds with their summarized SAR.

**Scheme 24:**
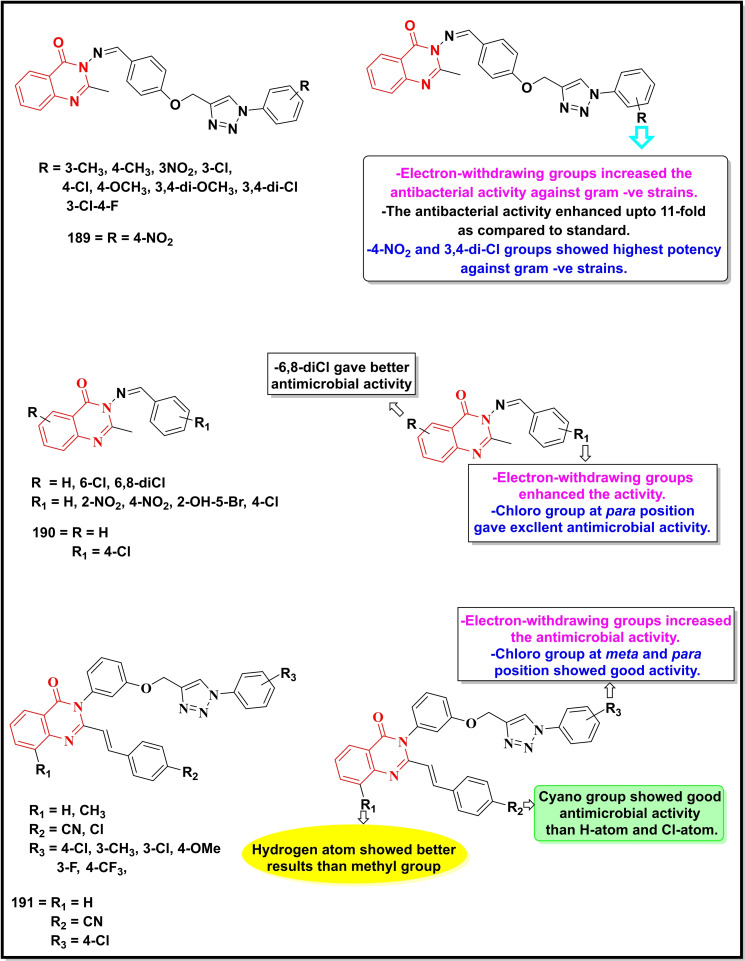
Showing general structures of tested quinazolin‐4‐ones and the potent antimicrobial compounds with their summarized SAR.

**Scheme 25:**
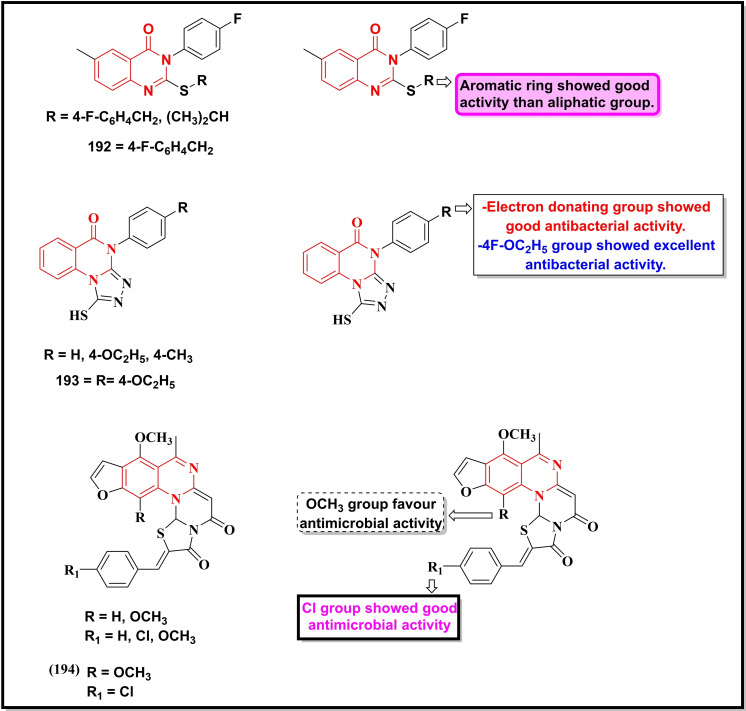
Showing general structures of tested quinazolines/quinazolin‐4‐ones and the potent antimicrobial compounds with their summarized SAR.

**Scheme 26:**
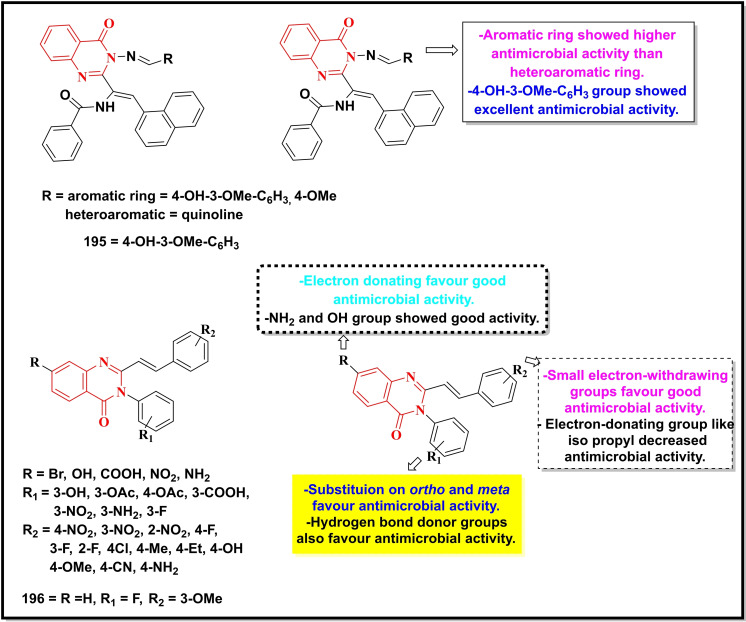
Showing general structures of tested quinazolines/quinazolin‐4‐ones and the potent antimicrobial compounds with their summarized SAR.

Schemes 22, 23, 24, 25, 26 depict the structures of some selected quinazoline/quinazoline‐4‐one derivatives with potential reported antimicrobial activities while their corresponding MIC values zones of inhibition (≥15 mm) against one or more bacterial and fungal strains (where applicable) are depicted in Table 2. These quinazolines fall into several classes of substituted quinazolines ranging from simple to more complex dimers. Predominantly, these quinazolines were substituted at N‐1, C‐2 and N‐3.

**Table 2:** Antimicrobial activity of quinazoline compounds. MIC values are shown in brackets while the Zone of inhibition (ZI) values are outside the brackets (where available).

Comp. No.	Gram +ve		Gram ‐ve			Fungi							
	*B. S*	*S. A*	*E. C*	*S. T*	*K. P*	*D. H*	*N. Sp*.	*A. N*	*B. T*	*R. N*	*F.N*	*C.A*	*C.N*
	ZI (mm), (MIC)												
171	(6.25)	(12.5)	–	–	–	(12.5)	–	–	–	–	–	–	
172	(31.2)	(62.5)	(7.8)	–	–	–	–	–	–	–	–	–	
173	55	49	67	–	64	–	50	62	55	52	–	–	
174	59	51	71	–	66	–	65	72	68	65	–	–	
175	61	53	73	–	70	–	67	75	69	67	–	–	
176	68	51	65	–	72	–	57	67	61	63	–	–	
177	69	50	71	–	77	–	54	63	59	65	–	–	
178	–	–	>20	–	–	–	–	–	–	–	–	–	
179	–	–	>20	>20	–	–	–	–	–	–	–	–	
180	–	–	–	>20	–	–	–	–	–	–	–	–	
*181	–	–	–	–	–	–	–	–	–	–	62(300)	–	–
*182	–	–	16(3.75)	–	–	–	–	28.57(15)	–	–	42 (15)	25 (7.5)	–
183	–	–	–	–	–	–	–	–	–	–	–	(16)	(8)
184	–	(20)	(23)	–	–	–	–	–	–	–	–	–	–
^#^185	–	(0.016)	(1)	–	(64)	–	–	–	–	–	–	–	–
^#^186	–	(0.29)	(8)	–	(>64)	–	–	–	–	–	–	–	–
187	–		(0.31)	–	–	–	–	–	–	–	–	–	–
188	–	(0.063)	(32)	–	–	–	–	–	–	–	–	–	–
189	–	33	14	–	–	–	–	–	–	–	–	–	–
190	(128)	(32)	(128)	–	–	–	–	–	–	–	–	–	–
191	–	(0.5)	(>64)	–	–	–	–	–	–	–	–	–	–
192	15(25)		25(12.5)	–	–	–	–	–	–	–	–	–	–
193	(5.2)	(2.6)	(5.2)	–	–	–	–	–	–	–	–	(12.5)	–
194	–	(4)	(2)	–	(1)	–	–	–	–	–	–	–	–
195	12	17	15	–	–	–	–	–	–	–	–	20	–
196	–	(0.004)	–	–	–	–	–	–	–	–	–	–	–

MIC=Minimum inhibitory concentration in μg/mL, *mg/ml, and ^#^μM B. S=Bacillus subtilis; S. A=Staphylococcus aureus; E. C=Escherichia coli; S. T=Salmonella typhimurium; K. P=Klebsiella pneumonia; D. H=Dreschlera haloids; N. Sp=Nigrospora sp; A. N=Aspergillus niger; B. T=Botrydepladia thiobromine; R. N=Rhizopus nigricum; F.N=Fusarium oxysporum; C.A= Candida Albicans; C.N= Candida neoformans.

Compounds **171–180** (Scheme 1.31) contain substitution at C‐2 and N‐3 except for compound **171** which is substituted at N‐1, C‐2, and N‐3 with N,N‐dimethylaminomethyl, thiocarbonyl and phenyl groups respectively, showed good activity against *B. subtilis, S. aureus* and *Dreschlera haloids* with MIC values of 6.25 and 12.5 μg mL^−1^ respectively.^[149]^ Compound **172** bearing a functionalized isatin group at C‐2 displayed a strain‐specific activity against *E. coli* with a MIC value of 7.8 μg mL^−1^.^[150]^ Five compounds of the same series (**173–177)** were generally active against multiple strains (both bacterial and fungal) particularly against *E.coli*, whereas the remaining compounds (**178–181**) were moderately active against one or two strains. Quinazolin‐4‐one derivative bearing substituted pyrazole moiety at C‐2 (**182**) was reported to be selective against three fungal strains, *C. Albicans* (ZI=28.5 mm), *A. niger (ZI=42 mm)* and *F. oxysporum* (*ZI=25 mm*) with a moderate activity against a bacterial strain, *E. coli* (16 mm).^[151]^

Compounds **183–188** are disubstituted quinazolin‐4‐ones and were predominantly explored at C‐2 and C‐6 positions.^[152]^ These compounds were active against two bacterial strains (*S. aureus* and *E. coli*) with the two prototypes, **185** (MIC=0.016 μM) and **186** (MIC=0.29 μM) showing the highest activity against *S. aureus*. Compound **187** displayed its selectivity towards *E. coli* (MIC=0.31 μg mL^−1^) while its counterpart **188** was active against *S. aureus* (MIC=0.063 μg mL^−1^).^[153]^

Quinazolin‐4‐one Schiff base when coupled with a 1,2,3‐triazole moiety (**189**) showed good activity towards *S. aureus* (ZI=33 mm) and *E.coli* (ZI=14 mm).^[154]^ Another Schiff base (**190**) without any additional pharmacophore showed moderate inhibition of three bacterial strains, *S. aureus*, *E.coli* and *B. subtilis* with MIC values between 32–128 μg/ml.^[155]^ The molecular hybrid of Quinazolin‐4‐one and 1,2,3‐triazole (**191**) showed potent activity (MIC=0.5 μg/ml) and selectivity towards *S. aureus*.^[156]^

A fluorinated quinazolinone (**192**) was moderately active (MIC=12.5 μg/ml) against *E. coli*,^[157]^ whereas its fused heterobicyclic analogues (**193–194**) displayed promising activity against *S. aureus* and *E. coli* with MIC <3 μg/ml.[^158^, ^159^] Compound **195** was the moderate inhibitor of *S. aureus*, *E.coli* and *B. subtilis (ZI 12–17 mm)*.^[160]^ *Finally, the sulphonamide analogue of* Quinazolin‐4‐one (**196**) displayed excellent inhibition of *S. aureus* with a MIC value of 0.004 μg/ml.^[161]^

## 4 Conclusion and Future Perspective

Quinazoline is a part of several biologically active natural products and synthetic compounds. Considering its medicinal relevance, many synthetic approaches have been employed to prepare its structural analogues in the past decades. This review focuses on different synthetic approaches including metal catalyst, non‐metal catalyst and miscellaneous catalysts used by researchers worldwide for the synthesis of different quinazoline/quinazolin‐4‐one analogues followed by a systematic account of their anticancer and antimicrobial activities (where applicable) in the past eight years.

Apart from the use of metal catalysts such as first and second‐row transition metals (e. g. Cu, Ni, Ru, Rh, etc.) in polar solvents under heating condition, several researchers also emphasized on the use of non‐metal synthesis of these heterocycles by employing different organic reagents alone or in combinations including greener reaction conditions (Table 3). The pyrimidine ring of quinazoline/quinazolin‐4‐one scaffolds was preferentially targeted for introducing new chemical moieties, particularly at C‐2 and N‐3 alongside some publications showing the exploration of C‐4 (in the case of quinazoline), C‐6 and C‐8 as well particularly when designing new antimicrobial and anticancer agents. Furthermore, the SAR analysis revealed that the appropriate placement of electron‐withdrawing, electron‐donating on these pharmacophoric units played a crucial role in the development of potent anticancer and antimicrobial agents. This information can serve as a guide for medicinal chemists to select suitable functional groups in order to design and develop more effective and safer molecules for the treatment of cancer and microbial infections.

**Table 3:** Summarised metal and non‐metal catalyst synthesis of quinazoline/quinazolin‐4‐ones.

Metal catalyst								
S.No.	Author^R^	Starting Material	Product	Catalyst	Additive	Solvent	Reaction conditions (Temperature/Time/Energy source)	Yield (%)
1	Hao *et al*. (2016)^[32]^	o‐alkenylphenyl carbodiimides, isocyanides	4,5‐dihydroimidazo[1,5‐a]quinazolines	CuI	K_3_PO_4_	THF	Reflux	60–85
2	Mahdavi *et al*. (2016)^[23]^	Arylmethanamines, isatoic anhydride	2‐substituted quinazolin‐4‐ones	CuBr	K_2_CO_3_	DMSO	120 °C, 8 h	66–77
3	Lei *et al*. (2016)^[33]^	*N*‐sulfonyl‐1,2,3‐triazoles, substituted 1,2‐benzisoxazoles	2,4‐disubstituted quinazolines	Rh_2_(esp)_2_	DBU	1,2‐DCE	160 °C, 5 min	35–80
4	Wang *et al*. (2016)^[34]^	Schiff bases and dioxazolones	Multisubstituted quinazolines	[Cp*RhCl_2_]_2_ and AgBF_4_	NaOAc	DCE	60 °C	72–98
5	Wang *et al*. (2016)^[35]^	different imidates and alkyl azides	2,4‐disubstituted quinazolines	[Cp*RhCl_2_]_2_	CuI and AgBF_6_	PhCl	90 °C, 16 h	36–90
6	Vidyacharan *et al*. (2016)^[36]^	Isocyanides, aromatic amines, Sec. amides	2‐amino‐substituted‐4(3*H*)‐quinazolinones	Pd(OAc)_2_	O_2_	DMSO	110 °C	65–78
7	Wang *et al*. (2016)^[37]^	benzimidates and dioxazolones	2,4‐disubstituted quinazolines	[Cp*RhCl_2_]_2_	AgBF_4_	DCE	50 °C, 5 h,	66–95
8	Wang *et al*. (2016)^[38]^	*N*‐sulfinylimines, benzimidates, dioxazolone	2,4‐disubstituted quinazolines	Cp*Co(CO)I_2_	AgNTf_2_	DCE	120 °C, 16 h	27–85
9	Bingi *et al*. (2016)^[162]^	anthrilamide and 1,3‐cyclic dione	tricyclic quinazoline	TsOH.H_2_O	–	m‐Xylene	150 °C, 8 h,	78–87
10	Liu *et al*. (2016)^[39]^	isatins and 2‐bromopyridine	pyrido‐fused quinazolinone	Cu(OAc)_2_⋅H_2_O	NaHCO_3_	DMF	120 °C, 2 h,	75–93
11	Wang *et al*. (2017)^[40]^	simple ketoximes and 1,4,2‐dioxazol‐5‐ones	Quinazoline N‐oxides	[Cp*RhCl_2_]_2_, Zn(OTf)_2_	HOAc	TFE	80 °C, 12 h,	55–91
12	Xu *et al*. (2017)^[41]^	CO_2_, isocyanide, and 2‐iodoanilines	quinazoline‐2,4‐(1H,3H)‐diones	PdCl_2_, PPh_3_,	DBU	MeCN	80 °C, 12 h,	42–95
13	Mampuys *et al*. (2017)^[42]^	2‐bromoanilines, carbon dioxide and isocyanides	*N*3‐substituted quinazoline‐2,4(1*H*,3*H*)‐diones	Pd(OAc)_2_	Ligand, Cs_2_CO_3_	1,4‐dioxane	80 °C, 7 h,	49–94
14	Liu *et al*. (2017)^[43]^	arenes and 2‐aminopyridines	pyrido‐fused quinazolinone	Cu(OAc)2	Air	DMSO	100 °C, 4 h,	62–84
15	Dubey *et al*. (2018)^[44]^	2‐halobenzoic acids and amidines	quinazolinones	Cu(OAc)2	Glucose, Cs_2_CO_3_	2‐MeTHF	rt, 24 h,	58–91
16	Prakash *et al*. (2018)^[45]^	2‐phenyldihydrophthalazinediones and alkynes	2,4‐disubstitutedquinazoline	[RuCl_2_(p‐cymene)_2_], dppp	Cu(OAc)_2_⋅H2O, K_2_CO_3_	AmOH	90 °C, 8 h	62–80
17	Majumdar *et al*. (2018)^[46]^	Alcohol and 2‐aminobenzamide	2,3‐dihydroquinazoline	Fe_3_O_4_−CND (10)	TBHP	Toluene	80 °C, 16 h	63–94
18	Hu *et al*. (2018)^[47]^	2‐aminobenzonitriles, aldehydes, and arylboronic acids	2,4‐disubstitutedquinazoline	Pd(acac)_2_	TfOH	DMF	80 °C, 24 h	60–91
19	Zhang *et al*. (2018)^[48]^	2‐(quinazolinone‐3(4*H*)‐yl)benzonitriles with arylboronic acids	2‐(4‐arylquinazolin‐2‐yl)anilines	Pd(OAc)2, 2,2’‐bipyridine	TsOH⋅H2O	Toluene	80 °C, 16 h	51–92
20	Wang *et al*. (2018)^[49]^	benzonitriles and 2‐ethynylanilines	2,4‐disubstitutedquinazoline	Cu(OAc)_2_	t‐BuOK	DMSO	120 °C, 16 h	41–88
21	Chen *et al*. (2018)^[50]^	2‐alkylamino Benzonitriles and organometallic reagents	4‐substitutedquinazoline	FeCl_2_	*t*BuOOH	DMSO	25 °C, 18 h	43–84
22	Parua *et al*. (2018)^[51]^	2‐aminobenzylamine with benzyl alcohol	2‐substitutedquinazoline	[Ni(MeTAA)]	KO^t^Bu	xylene	100 °C, 24 h	30–85
23	Zhu *et al*. (2018)^[52]^	arylboronic acids with N‐(2‐cyanoaryl)benzamides	2,4‐disubstitutedquinazoline	Pd(OAc)2, 2,2’‐bipyridine	TFA	THF	80 °C, 24 h	23–99
24	Ke *et al*. (2018)^[53]^	2‐halobenzoic acids and amidines	2‐substitutedquinazoline	CuCl_2_	NaOH	water	120 °C, 20 min, MW	50–93
25	Arachchige *et al*. (2019)^[54]^	2‐aminophenyl ketones and amines	2,4‐disubstitutedquinazoline	[(C_6_H_6_)‐(PCy_3_)(CO)RuH]+BF_4_,−4‐(1,1‐dimethylethyl)‐1,2‐ benzenediol	–	1,4‐dioxane	140 °C, 20 h	53–87
26	Wang *et al*. (2019)^[55]^	2‐arylindoles and dioxazolones	Indolo[1,2‐c]quinazolines	[Cp*RhCl_2_]_2_/AgSbF_6_	LiOAc	DCE	140 °C, 20 h	54–97
27	Ghorai *et al*. (2019)^[56]^	Triynes or tetraynes and nitrile	2,4‐disubstitutedquinazoline	AgSbF_6_	–	–	120 °C, 12 h	48–95
28	Jiang *et al*. (2019)^[57]^	Indols and *α*‐oxocarboxylic acids	Indolo[1,2‐ *a*]quinazolines	Pd(OAc)_2_	(NH_4_)_2_S_2_O_8_	diglyme	78 °C, 12 h	35–82
29	Sikari *et al*. (2019)^[58]^	2‐Bromobenzylamine and benzamide	2‐substitutedquinazoline	[NiII(L_3_)_2_]	NaOtBu	DMF	70 °C, 24 h	29–78
30	Xiang *et al*. (2019)^[59]^	*N*‐arylamidine and cyclopropenone	4‐ethenyl quinazolines	[Cp*RhCl_2_]_2_	AgSbF_6_	DCM	100 °C, 36 h	44–87
31	Chakrabarti *et al*. (2019)^[60]^	benzene‐1,2‐diamine and propane‐1,2‐diol	2‐substitutedquinazoline	Ir (III) complexes	KOH	H_2_O	100 °C, 24 h	60–94
32	Pathare *et al*. (2019)^[61]^	2‐azidobenzaldehyde, isocyanide, and hydroxylamine hydrochloride	Quinazoline 3‐oxides	Pd(OAc)_2_	–	Toluene	rt, 4 h	71–92
33	Biswas *et al*. (2019)^[62]^	carbon dioxide, isocyanides, and 2‐iodoaniline	quinazoline‐2,4(1H,3H)‐dione	Pd(II)EN@GO	DBU	MeCN	80 °C, 10 h	55–94
34	Pan *et al*. (2019)^[63]^	2‐(2‐aminophenyl)quinazolin‐4(3*H*)‐one and 2‐(2‐phenylethynyl)benzaldehyde	Fused quinazoline	AgOTf	–	DMSO	100 °C, 10 h	78–89
35	Yang *et al*. (2019)^[64]^	Amino benzamide and 3‐methyl‐1,4,2‐dioxazol‐5‐one	2,3‐disubstitutedquinazoline	[Cp*Co(CO)I_2_], Zn(OAc)_2_	AgNTf_2_	DCE	120 °C	52–99
36	Das *et al*. (2019)^[65]^	2‐aminobenzyl alcohol and benzonitrile	2‐substitutedquinazoline	NNS−Mn(I) complexes	^t^BuOK	xylene	140 °C, 30 h	86
37	Sun *et al*. (2020)	3‐aryl‐2H‐azirines with anthranils	(quinazolin2‐yl)methanone	Cu(OAc)_2_, AgSbF_6_	AcOH	DCE	100 °C, 12 h	43–72
38	Kerdphon *et al*. (2020)	Anthrilamide and primary alcohol	2,3‐dihydroquinazoline	Cu(OAc)_2_.H_2_O	Cs_2_CO_3_, O_2_	–	110 °C, 12 h	48–93
39	Mondal *et al*. (2020)	1,2‐Diols with 1,2‐Diaminobenzene	2‐subsitutedquinazoline	Mn(CO)_5_Br	KO^t^Bu	Toluene	130 °C, 36 h	58–81
40	Hu *et al*. (2020)^[69]^	aryl‐oxazoline and Tosyl chloride	2,3‐disubstitutedquinazoline	Cu(OAc)_2⋅_H_2_O	DMAP	DCE	120 °C, 10 h	43–86
41	Malasala *et al*. (2020)^[70]^	Aldehydes, aq. Ammonia, and 2‐bromobenzoic acid	2‐subsitutedquinazoline	CuI	O_2_	DMSO	100 °C, 10 h	70–82
42	Xu *et al*. (2020)^[71]^	Ethyl benzimidate and *N*‐methoxybenzamide	4‐oxy‐substituted quinazoline	[RhCp*Cl_2_]_2_	AgBF_4_	DCE	120 °C, 3 h	42–95
43	Tao *et al*. (2020)^[72]^	2‐aminobenzamide or 2‐aminobenzonitrile, Mo(CO)6 and aryl bromides	2‐substitutedquinazoline	PdCl_2_, BuPAd_2_	DBU	DMF	120 °C, 24 h	40–95
44	Philips *et al*. (2021)^[73]^	2‐amino‐*N*‐phenylbenzamide and *D*‐glucose	3‐substitutedquinazoline	CuBr	–	DMSO	120 °C, 12 h	44–91
45	Balaji *et al*. (2021)^[74]^	benzyl alcohols and 2‐aminobenzamide	Quinazolin‐4(3H)‐ones	[PdCl_2_(PPh_3_)_2_]	KOH	Xylene	110 °C, 24 h	54–91
46	Fan *et al*. (2021)^[75]^	ethyl benzimidate with *N*‐methoxyamide	2,3‐disubstitutedquinazoline	[Cp*IrCl_2_]_2_	AgBF_6_	DCE	120 °C, 4 h	72–94
47	Charpe *et al*. (2021)^[76]^	4‐chloro‐N‐phenylbenzimidamide and phenylacetylene	2,3‐disubstitutedquinazoline	CuCl_2_	O_2_	DCM/MeOH (2 : 1)	rt, 22 h, Blue LED	63–78
48	Bhattacharyya *et al*. (2022)^[77]^	Secondary alcohols and 2‐aminobenzyl alcohol	2‐substituted quinazolines	Ru‐catalyst	KO^t^Bu	Toluene	120 °C, 12 h	81–96
49	Pal *et al*. (2022)^[78]^	benzyl alcohols and 2‐aminobenzamide	quinazolin‐4(3H)‐one	Mn‐catalyst	NaO^t^Bu	Xylene	140 °C, 36 h	34–86
50	Wu *et al*. (2022)^[79]^	2‐aryl‐1H‐imidazoles and 1,4,2‐dioxazol‐5‐ones	imidazo[1,2‐c]quinazoline	[Cp*Co(CO)I_2_]	AgBF_6_	DCE	120 °C, 24 h	44–76
51	Martos *et al*. (2022)^[80]^	2‐acylanilines and benzylamines	2,4‐disubstitutedquinazolines	IBIS	Air	–	130 °C,22 h	29–84
52	Sarieh *et al*. (2023)^[81]^	5‐chloro‐2‐aminobenzophenone, aryl benzaldehydes, NH_4_OAc	Trisubstituted quinazolines	GO@Fe_3_O_4_@TRMS@HBPB@Cu	‐	–	Solvent free, 45 °C	88–95
53	Chikkagundagal *et al*. (2023)^[60]^	1‐aryl‐ and 2‐aryl‐1,2‐dihydro‐3*H*‐indazol‐ones, aldehydic N‐tosylhydrazones	1,2‐di(hetero)aryl‐and 2,3‐di(hetero)aryl‐2,3‐dihydroquinazolin‐4(1*H*)‐ones	Pd(OAc)_2_	Cs_2_CO_3_	Toluene	100 °C, 4 h	85–91
54	Wang *et al*. (2023)^[83]^	4‐phenylquinazolines, aromatic aldehydes	Quinazoline‐tagged aromatic ketones	Pd(OAc)_2_	Tert‐butyl hydroperoxide	PhCl	110 °C, 36 h	40–87
55	Natália M. Moreira *et al*. (2023)^[84]^	α‐substitutedβ‐nitrostyrenes,4‐methylquinazolines	Pyrrolo[1,2‐*c*]quinazolines	Cu(OAc)_2_	‐	–	‐	39–89

Non‐metal catalyst								
56	Chen *et al*. (2016)^[85]^	Anthranillic acid and formamide	quinazolin‐4(3H)‐one	–	–	DMF	Heating	NR
57	Hou *et al*. (2016)^[22]^	5‐Bromoanthranillic acid and formamide	6‐bromopyrido[2,3‐d]pyrimidin‐4‐ol	–	–	DMF	Heating	74
58	Kraege *et al*. (2016)^[18]^	Anthranillic acid and formamide	quinazolin‐4(3H)‐one	–	–	–	Reflux	73
59	Hei *et al*. (2016)^[11]^	Substituted anthranillic acid and formamidine acetate	Substituted quinazolin‐4(3H)‐ones	‐	‐	‐	Heating	74–96 %
60	Qin *et al*. (2016)^[86]^	Anthranilic methyl ester and formamidine acetate	Fused quinazolin‐4(3H)‐one and quinazoline analogues	–	–	Ethanol	Reflux	63–82 %
61	Yin *et al*. (2016)^[87]^	Anthranilic methyl ester and formamidine acetate	Fused quinazolin‐4(3H)‐one	–	–	Ethanol	Reflux	82 %
62	Ding *et al*. (2016)^[88]^	Anthranilic acid, acetic anhydride, Ammonia	2‐methyl‐quinazolin‐4(3H)‐one	–	–	Acetic anhydride	Reflux	70 %
63	Navale *et al*. (2016)^[90]^	Anthranilic acid, acetic anhydride, hydrazine	2‐methyl‐3‐amino‐quinazolin‐4‐one	–	–	Methanol	Heating	‐
64	El‐Serwy *et al*. (2016)^[89]^	Anthranilic acid, formamide, chloroacetyl chloride, hydrazine	2,4‐disubstituted‐ quinazolin‐4‐one	–	–	Ethanol	Reflux	65–75
65	Baluja *et al*. (2016)^[91]^	Chalcone derivatives of dihydryonaphthalene, 5‐amino‐pyrazole derivative	Pyrazoloquinazolines	–	–	n‐butanol	Reflux	71–79
66	Bonacorso *et al*. (2016)^[163]^	1‐methoxy‐2‐trifluoroacetyl‐3,4‐dihydro‐naphthalene, amidine salts	Dihydrobenzoquinazoline derivatives	–	NaOH	CH_3_CN	Heating	40–70 %
67	Nasrullayev *et al*. (2016)^[92]^	Anthranilic acid, β ‐lactam	Deoxyvasicinone	–	POCl_3_	‐	‐	70 %
68	Saad *et al*. (2016)^[93]^	2‐cyano‐4‐nitrophenyl‐*N*, *N*‐dimethylformamide, Anilines	4‐substituted quinazolines	–	–	Acetic acid	Heating	80–92 %
69	El‐Messery *et al*. (2016)^[94]^	anthranilic acids, substituted isothiocyanates	2‐mercaptoquinazoline‐4‐ones	–	TEA	Ethanol	Reflux	–
70	Mohamed *et al*. (2016)^[95]^	anthranilic acids, Aryl isothiocyanates	2‐mercaptoquinazoline‐4‐ones	–	TEA	Ethanol	Reflux	85–90
71	Appani *et al*. (2016)^[96]^	Anthranilates, substituted dithiocarbamic acids	2‐thioquinazoline derivatives	–	–	Ethanol	RT	–
72	Thakare *et al*. (2016)^[17]^	Urea, anthranilic acid	Quinazoline‐2,4‐diol	–	–	–	Heating	–
73	Krysko *et al*. (2016)^[97]^	Urea, 2‐aminophenyl‐phenyl methanone	Disubstituted Quinazoline‐2‐one	–	–	–	Heating	7 %
74	Aravind *et al*. (2016)^[98]^	Anthranilamide, benzaldehyde	2‐phenylquinazolin‐4(3H)‐one	Acetic acid	‐	DMSO	Heating	NR
75	Paumo *et al*. (2016)^[19]^	Anthranilamide, Aryl benzaldehydes	2‐aryllquinazolin‐4(3H)‐ones	I_2_	–	Ethanol	Reflux	86–93
76	Ghosh *et al*. (2016)^[99]^	Anthranilamide, Phenylacetaldehyde, DDQ	2‐benzyl analogues of quinazolin‐4‐one	Tartaric acid	dimethylurea (DMU)	‐	RT	78–82 %
77	Zhou *et al*. (2016)^[100]^	Isatin, guanidine	2,4‐disubstituted quinazolines	‐	NaOEt	CH_3_OH	Heating	65–75 %
78	Esvan *et al*. (2016)^[101]^	Isoquinoline aldehyde,Guanidine carbonate	10‐nitropyrido[3,4‐g]quinazolin‐2‐amine	–	H_2_CO_3_	DMF	MW irradiation	49
79	Jin *et al*. (2016)^[15]^	2‐aminobenzohydrazides and 2‐formylbenzoic acid	5H‐phthalazino[1,2‐b]quinazoline and isoindolo[2,1‐a]quinazoline derivatives	I_2_	–	Ionic liquid	Heating	81–90
80	Zhang *et al*. 2016)^[13]^	2‐aminobenzohydrazide and 1,7‐dichloro‐heptan‐4‐one	Pyridazino[6,1‐b]pyrrolo[1,2‐a]quinazolin‐9(1H)‐ones	I_2_	NaHCO_3_	CH_3_CN	Reflux	79–91
81	Zhang *et al*. (2016)^[14]^	Methyl 2‐amino‐5‐methoxy‐4‐(3‐(piperidin‐1‐yl)propoxy)benzoate, triethyl orthoformate	6‐methoxy‐7‐(3‐(piperidin‐1‐yl)propoxy)quinazolin‐4(3H)‐one	–	CH_3_COONH_4_	CH_3_OH	Reflux	NR
82	Desroches *et al*. (2017)^[102]^	2‐chloro‐N‐(2‐cyanophenyl)‐2,2‐difluoroacetamide	2‐Chlorodifluoromethylquinazolin‐4(3H)‐one	–	30 % NaOH, H_2_O_2_	EtOH/H_2_O	0° C to RT, Stirring	80
83	Marzaro *et al*. (2016)^[103]^	carbamate‐protected anilines, hexamethylenetetramine	3‐substituted quinazolines	–	K_3_Fe(CN)_6_, 10 % KOH	CF_3_COOH	MW	–
84	Agbo *et al*. (2016)^[104]^	2‐acetamido‐5‐bromobenzamide	6‐bromo‐2‐methylquinazolin‐4‐one	–	5 %NaOH	EtOH	Reflux	89
85	Shiri *et al*. (2017)^[164]^	2‐aminobenzimidazole and 2‐chloro‐3‐quinolinecarboxaldehyde	Fused quinazoline	K_2_CO_3_	–	DMF	MW, 3–5 min Or 120 °C, 180 min	83–90
86	Pandya *et al*. (2017)^[105]^	2‐aminibenzophenone and aminonitrile	2‐aminoquinazoline	*p*‐TSA	–	DMF	110 °C, 12 h	75–80
87		2‐aminibenzophenone and cyclic 2‐aminonitrile	cyclic 2‐aminoquinazoline	KOtBu	–	DMF	110 °C, 12 h	75–81
88	Ambethkar *et al*. (2018)^[106]^	methyl ketones and 2‐(2‐Aminophenyl) Benzimidazole	Benzoimidazo Quinazolinones	I_2_	–	DMSO	110 °C, 6 h	52–83
89	Saikia *et al*. (2018)^[107]^	2‐aminobenzophenones/acetophenones and anthranilic acids with nitriles.	2,4‐disubstitutedquinazoline	TMSOTf	–	–	110 °C, 6 h, MW	72–81
90	Mukhopadhyay *et al*. (2018)^[108]^	2‐amino‐*N*‐[4‐(*tert*‐butyl)phenyl]‐benzamide	3‐susbtitutedquinazoline	TBHP	Cs_2_CO_3_	CH3CN	80 °C, 10 h	38–86
91	Chen *et al*. (2018)^[109]^	Aniline, aldehyde, ammonium chloride	2,4‐disubstitutedquinazoline	PhCl	O_2_	DMSO	150 °C, 12 h	40–95
92	Chatterjee *et al*. (2018)^[110]^	2‐Aminobenzylamines and *α*,*α*,*α*‐trihalotoluenes	2‐aryl quinazolines	NaOH or DABCO	O_2_	H_2_O	100 °C, 16 h	52–81 or 61–78
93	Sharma *et al*. (2018)^[111]^	2‐aminobenzamide	quinazolines and quinazolinones	Oxalicacid		1,4‐Dioxane	120 °C, 14 h	82–89
94	Bahadorikhalili *et al*. (2019)^[112]^	isatoic anhydride, amines, and sulfur	2‐Thioxo‐2,3‐dihydroquinazolin‐4(1*H*)‐one Derivatives	potassium *tert*butoxide	CHCl_3_	1,4‐dioxane	60 °C	58–79
95	Hada *et al*. (2019)^[113]^	isatoic anhydride and benzylamines	3‐substituted quinazolinone	GO (grephene oxide)	–	DMSO	130 °C, 24 h	64–84
96	Xie *et al*. (2019)^[114]^	2‐aminobenzamide	Fused quinazoline	(NH_4_)_2_S_2_O_8_	–	DMSO	60 °C, 2 h	21–94
97	Yan *et al*. (2019)^[12]^	2‐aminoacetophenones with isocyanates or isothiocyanates	3‐substituted 4‐methylene‐quinazolinthiones and 4‐methylenequinazolinones	NaOH	–	CH_3_CN	80 °C, 1.5 h	41–98
98	Garia *et al*. (2019)^[115]^	N‐pyridylindoles	fused quinazolinones	DIB	K_2_S_2_O_8_	CH_3_CN	80 °C, 2 h	60–82
99	Jatangi *et al*. (2019)^[116]^	2‐aminobenzophenones and phenylisothiocynate	*N*,4‐disubstituted quinazoline‐2‐amine	I_2_	NH_4_OAc	DMSO	50 °C, 2 h	87–95
100	Lee *et al*. (2019)^[117]^	2‐aminobenzamide	3‐substituted quinazolines	DABCO	K_2_S_2_O_8_	DMSO	160 °C, 2 h, MW	47–72
101	Trinh *et al*. (2020)^[118]^	2‐aminoaryl ketones, aldehydes, and ammonium acetate	2,4‐substituted quinazolines	H_2_O_2_	air	DMSO	60 °C, 6 h	61–89
102	Chen *et al*. (2020)^[119]^	2‐aminobenzamide and 2‐amino‐N‐methylbenzamide	quinazoline‐2,4‐diones	DMAP	(Boc)_2_O	CH_3_CN	MW, 30 min or rt, 12 h	57–92 or 53–82
103	Pariyar *et al*. (2020)^[120]^	2‐aminobenzamide and aldehydes or ketones.	2,3‐dihydroquinazolin‐4(1H)‐ones	L‐ascorbic acid	Oxone	Water	90 °C, 1 h	72–92
104	Gujjarappa *et al*. (2020)^[121]^	2‐aminobenzylamines and nitriles	2‐substituted quinazolines	Vit. B3	air	DMSO	110 °C, 24 h	70–91
105	Ramu *et al*. (2020)^[122]^	3‐ylideneoxindoles and tosyldiazomethane	pyrazolo‐[1,5‐c]quinazolines	K_2_CO_3_	–	Water/acetone	60 °C, 4 h	60–90
106	Chan *et al*. (2020)^[123]^	2‐aminobenzophenone and aldehyde	2,4‐disubstituted quinazolines	TMSOTf	HDMS	‐	150 °C, 0.5 h	81–92
107	Viji *et al*. (2020)^[124]^	2‐(1H‐pyrrol‐1‐yl) Aniline and lactic acid	pyrrolo[1,2‐a]quinoxalines, and quinazolinones,	TBHP	‐	DCE	80 °C, 10 h	52–84
108	Yuan *et al*. (2021)^[125]^	*N*‐(*o*‐tolyl)benzimidamide	2‐substituted quinazolines	–	air	DMSO	165 °C, 12 h	40–92
109	Arandhara *et al*. (2021)^[165]^	3‐(1‐hydroxy‐3‐oxoisoindolin‐2‐yl)‐N‐phenylpropanamide	Fused quinazoline	BF_3_OEt_2_	–	DCM	Rt, 0.25 h	91–97
110	Govindan *et al*. (2022)^[126]^	2‐aminobenophenone	N, N‐disubstituted 2,3‐ dihydroquinazolin‐4(1H)‐ones	NH_4_SCN	Piperidine	DMSO	120 °C, 24 h,	37–85
111	Zhao *et al*. (2022)^[127]^	methyl 2‐aminobenzoates or 2‐aminobenzamides using 2,2,2‐trichloroethyl imidates hydrochloride	2‐substituted quinazolines	NaOAc	–	DCM	Rt, 4 h	50–98
112	Yamamoto *et al*. (2022)^[128]^	o‐aminobenzylamines and benzylamines	2‐substituted quinazolines	4,6‐dihydroxysalicylic acid	BF_3_OEt_2_/O_2_	DMSO	90 °C, 48 h,	17–81

^R^ Reference.

However, several reported methods suffered from limitations due to harsh reaction conditions employed and limited substrate versatility. Hence, it is important to fine tune these reaction protocols to improve their reaction outcome, for example, by utilizing sterically hindered substrates and more suitable reagents to minimize the side reactions and improve optimal product yields. Additionally, eco‐friendly processes should be developed in accordance with the principles of green chemistry. Chemical modification of existing drugs bearing these scaffolds can be also considered by retaining their beneficial properties while addressing their limitations concurrently. Another important strategy to look at is to incorporate functional groups or structural fragments that enhance physicochemical properties and binding affinity to relevant target sites, thereby improving their biological activity, expanding the activity spectrum, and overcoming drug resistance overall. Additionally, developing structurally novel quinazoline and quinazolinone derivatives for potential medicinal applications can be another avenue to be explored by the synthetic chemists. These novel derivatives may operate through new mechanisms and display unique bioactivity. We hope that this review which is complementary to the previously published ones will update the relevant scientific community with the synthetic and medicinal aspects of this promising heterocycle for exploring it further in their drug design and drug synthesis endeavors.

## Conflict of Interests

The authors declare no conflict of interest.

## Biographical Information

*Neha Manhas completed her B Pharm from India before moving to South Africa to do her MSc degree in Chemistry from the Durban University of Technology (South Africa) in 2014, and then her Ph.D degree in Chemistry from the University of KwaZulu Natal (South Africa) in 2020. She is currently doing her PDF research in the Department of Chemistry at the Durban University of Technology. Her research interests involve the synthesis of novel heterocyclic scaffolds and their biological activity testing against cancer and microbial infections. She is also using different computational methods to support her experimental findings. She has published her research findings in top‐ranked medicinal chemistry journals with an international reputation and has presented her research work at both local and international research platforms*.

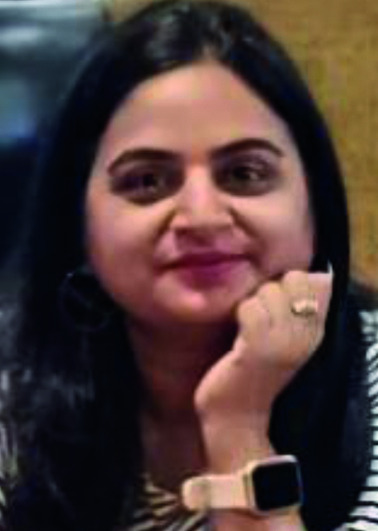

## Biographical Information

*Mr. Gobind Kumar received his Master's degree in Chemical Sciences from I. K. Gujral Punjab Technical University, Kapurthala, Punjab (India) in 2019. Currently, he is pursuing his PhD degree in the School of Chemistry and Physics at the University of KwaZulu‐Natal, Durban (South Africa), under the supervision of Prof. Parvesh Singh and co‐supervision of Prof. Gaurav Bhargava. His research interests include the greener synthesis of novel heterocyclic compounds and their testing as antitubercular and antidiabetic agents followed by their validation using Density functional theory and Molecular Docking calculations*.

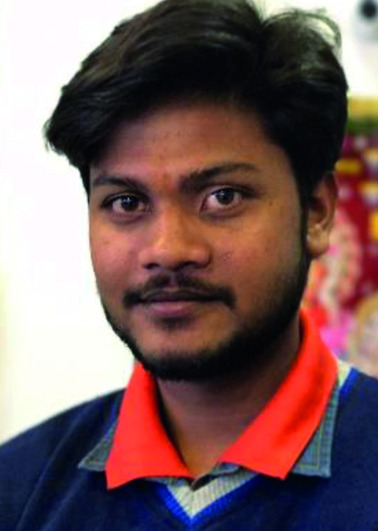

## Biographical Information

*Sanjeev Dhawan completed his doctoral degree (Chemistry) in 2018 under the supervision of Prof. Parvesh Singh from the School of Chemistry and Physics at the University of KwaZulu Natal followed by his PDF research in the School of Health Sciences in the same institution. His research area revolves around the synthesis of a variety of heterocyclic compounds, their characterization using different spectroscopic techniques followed by their biological testing against various cancers, and microbial strains. He has co‐authored around 25 publications in high‐impact journals of international repute and has presented his work at both national and international conferences*.

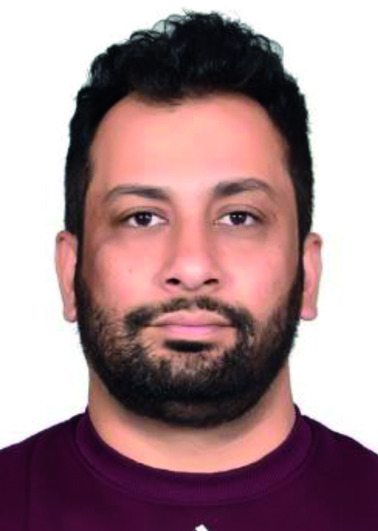

## Biographical Information

*Dr Makhanya holds a PhD degree in Chemistry. He is currently working as a Senior Lecturer in the Department of Chemistry at Durban University of Technology (South Africa). His research specialization is focusing on designing, synthesizing and characterization of new heterocyclic molecules including naphthyridines, pyrazoles, indole‐pyrazoles, quinolone, steroidal, thiazole, coumarin pyrazole and amino‐phosphonates. His research also involves the development of alternative methodology to afford efficient and rapid synthesis of new heterocyclic drugs using multicomponent reactions. The synthesis of such molecules is pursued for monotherapy or as a combination in treatment of life‐threatening diseases. This subject is highly topical most specially in modern drug discovery owing to undesirable side effects showed by current therapeutic drugs and emerging of new viruses like Covid‐19*.

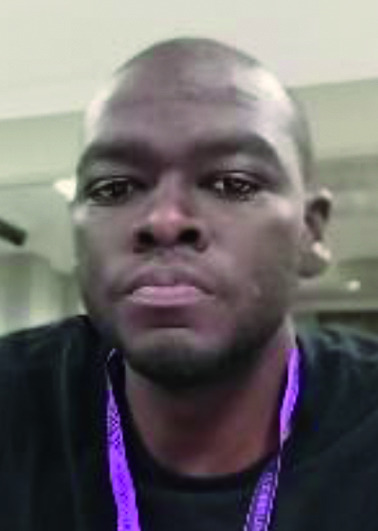

## Biographical Information

*Parvesh Singh is an Associate Professor (Organic Chemistry) in the School of Chemistry and Physics at the University of KwaZulu Natal, Durban (South Africa). His research includes the synthesis of heterocycles and molecular hybrids of different pharmacophoric assemblies to assess their medicinal potential against diabetes, cancer and bacterial infections including tuberculosis followed by their supplementation using different molecular modelling studies. He is an NRF‐Rated researcher and has been a recipient of top university/College research awards on several occasions. He has graduated around 30 postgraduate students including 4 PDFs and 2 Research Associates. He has published around 160 publications in peer‐reviewed journals including 4 books with reputed publishers and serving as Associate/guest editor and reviewer for several internationally reputed journals of organic synthesis and medicinal fields*.

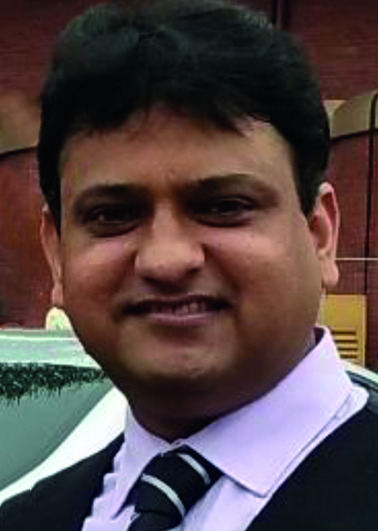

## Data Availability

Data sharing is not applicable to this article as no new data were created or analyzed in this study.
